# Kandinsky or Me? How Free Is the Eye of the Beholder in Abstract Art?

**DOI:** 10.1177/2041669519867973

**Published:** 2019-09-04

**Authors:** Doris I. Braun, Katja Doerschner

**Affiliations:** Abteilung Allgemeine Psychologie, Justus-Liebig-University, Gießen, Germany; Abteilung Allgemeine Psychologie, Justus-Liebig-University, Gießen, Germany; Department of Psychology & National Magnetic Resonance Research Center, Bilkent University, Ankara, Turkey

**Keywords:** art perception, abstract art, color adjustment, cultural differences, pictorial center

## Abstract

We investigated in “art-naïve” German and Chinese participants the perception of color and spatial balance in abstract art. For color perception, we asked participants (a) to adjust the color of a single element in 24 paintings according to their liking and (b) to indicate whether they preferred their version of the painting or the original. For spatial perception, we asked participants (a) to determine the “balance point” of an artwork and (b) to indicate their preferences for the original or left-right reversed orientation of previously seen and unfamiliar paintings. Results of the color experiments suggest that, even though the interactive task was of a rather open-ended nature, observers’ color adjustments were not random but systematically influenced by each painting’s color palette. Overall, participants liked their own color choices about as much as the original composition. Results of the spatial experiments reveal a remarkable consistency between participants in their balance point settings. The perceived lateral position of the balance point systematically affected the left-right orientation preference for a given painting. We conclude that “art-naïve” observers are sensitive to the composition of colors and spatial structures in abstract art and are influenced by their cultural backgrounds when experiencing abstract paintings.

## Introduction

Abstract art offers the possibility to study aesthetic sensitivity for color and composition, while attenuating strong influences of object or scene recognition (Montagner, Linhares, Vilarigues, & Nascimento, 2016; Oliva & Schyns, 2000; Tanaka, Weiskopf, & Williams, 2001; Witzel & Gegenfurtner, 2018). Because of the relatively high degree of independence from visual references to real world objects or scenes, abstract art is also called nonfigurative, objective or representational art, in contrast to the traditional accurate depiction of the visual world as found in the amazing realistic paintings of physical objects like in the still lives of the Dutch “Golden Age.” Many artists and art movements throughout the world contributed at different points in time to the development of a nonrepresentational painting style. While some artworks of Turner, Corot, Matisse, or Liang Kai are considered to be painted partially abstract, an untitled watercolor painting by Kandinsky from 1910 is often recognized as the first abstract artwork (also see “Über das Geistige in der Kunst” by Kandinsky, 1912).

To create an artwork is a complex process including continuous decisions with respect to the distribution, orientation and size of shapes, lines and structures, and the selection and placement of shades of color and contrasts. Whether an aesthetically pleasing composition is finally achieved depends on the delicate interactions between the different elements and colors. For example, adding or taking away a color can influence the whole chromatic composition of the artwork and change its former balance so that other parts of the painting “have” to be changed along with it in order to reach a new satisfying aesthetic solution. Creative processes like painting can take hours, days, or years; each painting speaks for the solution the artist has chosen at that moment in time and sometimes paintings are never finished or even get destroyed by the artist. Art works in museums represent—in a way—the artist’s solution reached after uncountable decisions during his/her work process.

On the other end of this creative process is the “receiver,” the person perceiving the artwork. While the receiver sees most of the times a “finished” artwork, artists reach their final solutions by selecting, for example, a certain color for an element after previous color choices and compositional changes of the painting. These decisions are based on years of experience, education, and training. If given the freedom, would a layperson “complete” an abstract painting the same way the artist would have? To what degree is the observer of art sensitive to this creative process? Do colors and composition “dictate” the color for a specific shape or element in a painting, as suggested by Kandinsky (1947)? And, are viewers of art able to perceive the compositional balance of the artwork? Here, we investigate the layperson’s sensitivity to the compositional properties of abstract art, the perception of color and weight, respectively, using two interactive tasks and two corresponding preference judgments.

What determines the aesthetic impression of an abstract painting are—to a large extend—its visual features, such as the specific selection and setting of paint colors and contrasts (Nascimento et al., 2017; Palmer & Schloss, 2010, 2011; Palmer, Schloss, & Sammertino, 2013; Tregillus & Webster, 2016); the balance of its different geometric elements, their distribution, and specific weights (Arnheim, 1954/1974; Beaumont, 1985; Fechner, 1876; Hübner & Fillinger, 2016; Koenderink, van Doorn, & Gegenfurtner, 2018; Locher, 2003; D. W. Ross, 1907; Wilson & Chatterjee, 2005); the degree of symmetry (Frith & Nias, 1974; Locher & Nodine, 1987; Tyler, 1999); its visual complexity (Berlyne, 1970; Berlyne & Ogilvie, 1974; Palmer et al., 2013); contrasts (Tinio, Leder, & Strasser, 2011; van Dongen & Zijlmans, 2017); size; and the specific painter style (brush stroke, gestures).

However, whether we find a painting aesthetically pleasing can also depend on nonvisual factors, such as age, education, knowledge, previous experience, implicit memory, context, and social interaction (Beke et al., 2008; Bogushevskaya, 2016; Leder, Belke, Oeberst, & Augustin, 2004; Silva, 2006; Witzel & Gegenfurtner, 2018). These artwork-independent factors tend to differ not just between individuals but also—at a larger scale—between socioeconomic classes or cultures, for example, engagement level with art or artistic style preference (Bennett & Silva, 2006), color preferences (Taylor, Clifford, & Franklin, 2013; Yokosawa, Yano, Schloss, Prado-Leon, & Palmer, 2010; Yokosawa, Schloss, Asano, & Palmer, 2016), or side preference for important objects (left or right; Wölfflin, 1928). We tested two art-naïve student groups from the Republic of China and Germany to investigate whether the cultural background influenced systematically the sensitivity to the compositional properties of abstract art. The results of our four experiments revealed that the color composition, in particular the hue variance of abstract artwork systematically influences participants when performing a color adjustment task. Although participants frequently arrived at different color solutions than the artist, they liked their own choices just as much. We established a link between the left-right orientation preference for an abstract painting and its perceived balance point. Overall, cultural differences emerged in these results.

## Methods

### Overview

We conducted four experiments, two concerning the perception of color and two concerning the spatial composition of abstract paintings for Chinese and European (German) participants. We first investigated how the chromatic composition of a painting influenced participants’ color choice for a single element in the same painting. Then we asked participants to indicate the “balance point” for each artwork and to designate their preference for original or mirror-reversed versions of familiar (from Experiments 1 and 2) and unfamiliar (new additional) abstract paintings. At the end, we asked participants to compare the painting with the target element colored according to artist’s or their own color choice and to indicate their preference.

### Stimuli

**Figure 1.**
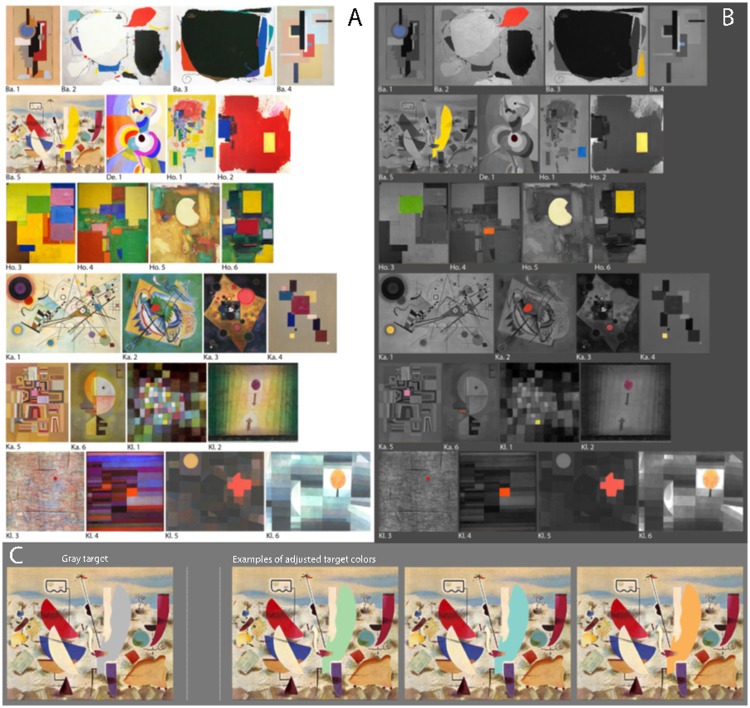
(a) Image of 24 paintings—5 of Willy Baumeister (Ba.), 1 of Sonja Delaunay (De.), 6 of Hans Hoffmann (Ho.), 6 of Wassily Kandinsky (Ka.), and 6 of Paul Klee (Kl.)—were used in our experiments. All paintings contained a suitable target, that is, a distinct element, colored by a single paint color, which was important for the color composition of the artwork. (b) Color, position, and relative size of the targets. (c) Example stimulus for the *target color adjustment* experiment. An image of a single painting appeared on the monitor with the selected target element shown in neutral gray. Participants had the task to adjust the color of the target such that they liked it for the painting by pressing one of six keys, four for color (red, green, yellow, and blue) and two for increasing and decreasing the luminance. See Methods section for further details. Movie 1 shows an animation of the adjustment task.

We selected 24 abstract artworks (see Figure 1(a), and Supplementary Table 1) from wikiart.org and used corresponding JPEG images of the highest resolution. For convenience, we often use the term “painting” when referring to the digitized JPEG image of the painting. To identify a given painting used in our experiments and in this study, we refer to each with the first two letters of the last name of the artist and a number, for example, Kl. 1 for the first artwork by Paul Klee on our table. For the two color experiments and the balance point experiment, we used five paintings Willy Baumeister, one by Sonja Delaunay, six by Hans Hoffmann, six by Wassily Kandinsky, and six by Paul Klee.

**Movie 1.** The movie shows an animation of the color adjustment task and illustrates the effect of different colors within the painting. The initial color of the target was neutral gray and key presses by the participant added more blue, yellow, red or green or made the color lighter or darker.

Our selection of the 24 artworks was based on the following criteria: They should be roughly from the same time period, the painting should contain a clearly defined element (we also call *target* from here onwards), painted in a single color, which was important for the balance of the whole color composition, but which was not the main or most dominant color of the painting. The target should also not be covered by any other element, structure, or line elements in the painting (see Figure 1(b)). The shape, size, and position of selected targets differed between paintings: The smallest target was 0.3% (Kl. 3), and the largest was 11% (Ho. 6) of the respective total painting area. Of the 24 target elements, 12 were rectangles (of these 4 were squares), 9 round shapes (7 circles and 2 ovals), 2 irregular shapes, and 1 triangle.

To allow for manipulation of the target color, we annotated the target element of each painting in Photoshop (Adobe Photoshop CC 2015) and used the resulting masks in the experiments. In both color experiments, all pixels within the target were set to have the same RGB values, effectively removing any potential information about paint and stroke structure and small hue variations. For the color adjustment task, the target colors of all 24 paintings were initially set to a neutral gray (RGB: 128 128 128). For the preference task, two versions of a painting were presented, one containing the target in the original (but averaged) color and one with the target color chosen by the participant. All targets had feathered boundaries and thus fit seamlessly into the original artworks, with no noticeable artifacts as shown for one of the paintings in Figure 1(c). Potential remains of the painting frame, or the passe-partout were cropped, and the artist’s signature removed by carefully recoloring the corresponding pixels with the surrounding color.

### Experimental Setup and Apparatus

During the experiments, a participant sat at a table in an illuminated room (daylight). Images were presented on a uniform black background at the center of the monitor and the position of the observer’s eye corresponded approximately to the height of the screen center. The monitor frame was covered and surrounded by a large white paper wall to induce the impression of a painting surrounded by a black frame hanging on a white wall. All stimuli were shown on a linearized computer monitor (Display ++, LCD, Cambridge Research Systems Ltd), driven at a 120-Hz refresh rate. At a viewing distance of 90 cm, the active screen area subtended 42.5 degrees visual angle horizontally and 24.45 degrees vertically on the participant’s retina, with a spatial resolution of 1920 × 1080 pixels this resulted in 45 pixels per degree visual angle. JPEGs were anti-gamma corrected before displaying them on the monitor. The experimental code was written in Matlab (The MathWorks, Inc.) using the Psychtoolbox (Kleiner et al., 2007).

### Experiments and Procedures

Each of the 40 participants conducted the four experiments in approximately 40 to 50 minutes in the same sequence but at his/her own pace with short breaks in between. Two experiments consisted of a color task: adjustment of the target color within each painting until a subjectively pleasing solution was reached and a preference choice between the painting with the adjusted or the one with the original target color. The other two experiments were concerned with the spatial composition of the paintings: the localization of the painting’s balance point and the preference for the painting in its original or mirror-reversed orientation. Experiments were run in the following order: *target color adjustment*, *balance point identification*, *laterality preference*, and *target color preference*. We will first describe the two color experiments, and then the two experiments querying on spatial aspects of the paintings.

#### Target color adjustment

**Figure 2.**
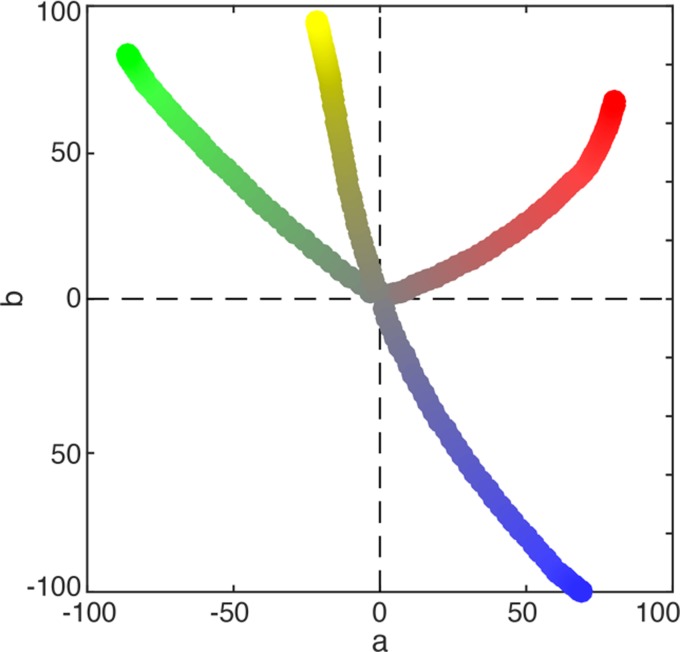
Color adjustment of the target in CIE L*a*b* space. In the experiment, participants could adjust the color of the target by navigating in RGB via the four arrow keys of the keyboard. This graph shows example trajectories of adjustments from neutral gray (RGB triplet [.5 .5 .5]) to the computer primaries and yellow (R [1 0 0], G [0 1 0], B [0 0 1], Y [1 1 0]). The brightness of the target color could be adjusted separately by two keys.

In this experiment, participants had the task to find the “paint” color they would like best for an element (target) in an abstract painting presented on the computer screen (Instruction: “Please choose a color you like for the gray element!”). At the beginning of each trial, the painting appeared with the target color set to neutral gray. Participants could then adjust the color of the target by navigating in RGB via arrow button presses (left: more red, right: more green, up: more yellow, and down: more blue). Figure 2 shows example trajectories (from gray (RGB triplet: [.5 .5 .5]) toward the computer primaries (R [1 0 0], G [0 1 0], B [0 1 1], Y [1 1 0]) in the widely used CIE L*a*b* space. The brightness of the target color could be adjusted by using the “Z” (darker) and “X” (brighter) keys. We told each participant to try as many colors as he/she liked and to stop, when he/she was contented with the adjusted color by pressing the space bar. The 24 paintings were presented in random order. The time in our experiments was unlimited and participants spend up to 40 minutes for 24 target color settings.

#### Target color preference (Kandinsky or me?)

Two versions for each of the 24 paintings were shown next to each other, one with the target in its original color and one with the target in the color chosen by the participant in the *target color adjustment* experiment. The participant had to compare both versions of the painting and to indicate his/her preference by pressing one of two assigned keys. (Instruction: “Which painting do you like better? The left or the right one?”). We replaced the pixels in the target element of the original painting with the mean RGB value of its original values such that it had the same smooth non-textured appearance as the target in the *target color adjustment* experiment. On each trial, the positions of the original and participants’ version of the painting (left or right side of the screen) were chosen randomly, as was the order of 24 paintings. This experiment was conducted at the end of the experimental session. The temporal break between the *target color adjustment* and *color preference* experiment was intended to reduce the participant’s memory his/her own color settings.

#### Balance point

When creating a painting, the artist also has to decide where to place areas of high complexity and strong luminance and color contrasts, where to leave more “open space” or unstructured areas, and how to balance the whole composition. Many factors determine where we perceive the compositional weight of a painting: the image geometry, the distribution of shapes, structures and uniform areas, color, and luminance contrasts. However, how we do it, which mechanisms allow us to evaluate the perceptual balance of complex pictures like paintings, is not at all clear (Koenderink et al., 2018). Participants were asked to determine the position and strength of the “balance point” or “center of gravity” for each of the 24 paintings of the *color adjustment* experiment. Together with each painting, a white rectangle of the same dimensions appeared on the right half of the screen. Using the computer mouse, participants had to position a small black dot within this white rectangle such that it corresponded to the perceptual balance point of the painting. They could also adjust the size of the black dot by pressing the two arrow keys such that it reflected the strength or importance of the balance point (Instruction: “Please move the black dot such that it corresponds to the center of gravity of the painting, and adjust the dot’s size to indicate its importance.”). Participants had the option to indicate that they did not perceive any clear balance point, or center, by pressing “x.”

#### Laterality preference

Participants indicated which one of two simultaneously presented versions of an artwork image they preferred—the original or its left-right reversed mirror image (Instruction: “Which painting do you like better? The left or the right one?”). To test for memory effects on the left-right orientation of the 24 paintings presented already in the *target color adjustment* and *balance point* experiments, we added a set of 24 new images of paintings of the same artists and their mirror images (see Supplementary Table 2). For this new set, we chose a similar number of artworks per artist painted in a similar style as in the first experiment. Thus, the stimulus set for this task contained 48 images of paintings presented simultaneously in their original orientation and their mirror-image versions. All 48 pairs of stimuli were presented in random order side-by-side with a gap of 2 degrees between them. Left-right positions of the original and flipped painting were randomized on each trial.

After the four experiments, participants completed a quick survey in which they were asked—among age, education, eye sight, what their favorite color was, how often they visited art museums, whether they had a favorite art stile, liked abstract art, which paintings they recognized, or whether they knew any of their artists. See Supplementary Text 1 and Supplementary Table 3.

### Participants

The European group consisted of 20 German students at Giessen University (age: 24.8 years; 5 males). The Chinese group consisted of 20 exchange students from the People’s Republic of China (age: 25.7 years; 6 males). All participants were art-naïve, that is, they did not study art or worked as artists or in any profession concerned with art. Both groups were also roughly education matched and had a similar social background. Participants were reimbursed for their participation, and before testing, they gave informed consent. All procedures were approved by the local ethics committee (Giessen University LEK 2013-0018) and were in accordance with the principles of the Declaration of Helsinki.

## Analysis

### Target Color Adjustment

#### Minimum distance

All color analysis was done in the CIE Lab color space, in which distances roughly correspond to perceptual measures (see, e.g., Fairchild, 2013). For each painting, we tested whether there was any systematic structure in the target color settings of our participants by comparing the average of the nearest (Euclidian) distances between the color choices of all participants to the average of the nearest distances computed on a uniform random sample of the same size (i.e., number of participants) taken from CIE Lab space (Smith, 2016), with the additional constraint that the random values had to be realizable within the RGB cube. To determine whether a given distance was significantly smaller than random, we used classic bootstrapping (Efron & Tibshirani, 1993) to determine the cutoffs corresponding to the 5th and 1st percentiles of the average distances randomly sampled from CIE Lab space. If the average distance computed from the participants’ color choices was below these cutoffs, we concluded that they were significantly structured and not randomly distributed.

#### Clustering

To efficiently compare target color settings with the color space of the respective original paintings, we clustered the CIE Lab pixel values of every JPEG image of each painting using the *k*-means algorithm (Matlab and Statistics Toolbox, The MathWorks, Inc.) with *k* = 20 and used the obtained centroid values in subsequent analysis. This algorithm works iteratively and minimizes the sum of distances (squared Euclidean distance) from each object to its cluster centroid, over all clusters. Painting clusters were computed without the target patch. For verification, we also repeated the clustering with a 3D Gaussian mixture model with 20 components (Nabney, 2002), which resulted in nearly the same cluster centroid values and cluster membership distributions (obtained by computing the posterior probability of each pixel given a Gaussian distribution). The target of each original painting was clustered independently with *k* = 5, and the mean CIE Lab value for each target computed from the respective five centroids. Targets were clustered to be able to assess the agreement of original target color space and target color settings of observers, as described here.

For a more global analysis, we determined the most frequently chosen hue category for both groups of participants. Category boundaries were set roughly to correspond to shades of blue [–2π/3, 5π/6], green [3π/7, 5π/6], yellow-orange [π/7, 3π/7], red [–π/3, π/7], and violet-purple [–π/3, –2π/3]. For a given painting, we determined the “winner” category along and the percentage of the participants, who selected a hue from this category. Occasionally, the most frequent target hue setting fell into two or three categories (Supplementary Figure 3).

#### Influence of painting hue variance on target hue variance

Each painting has its own range of paint colors and this range varies across artists and paintings. We were interested to know to what extend the color palette used for the painting restricted participants’ choices for the target color. For example, some paintings had a much smaller range of colors, for example, Kl. 2, or Ba. 1 compared to Ho. 6 or Ba. 3. To investigate this question, we converted CIE Lab values to polar coordinates L*C*h°, where h° corresponded to the hue angle and the radius C*_ab_ corresponded to chroma, and regressed— each group of participants—*h° variance* of the target settings onto *h° variance* of the paintings. Note that we computed the circular variance of *h°* using the Circular Statistics Toolbox for Matlab (The MathWorks, Inc.). A significant *R*^2^ implies that overall, participants’ target color choices were influenced by the gamut of colors they saw in the painting image. We conduct analogous analyses for luminance (CIE L*) and chroma settings (C*_ab_).

### Agreement of Painting Color Space and Target Settings

We tested if and how many of the participants’ color choices for the target were inside the space delineated by the 20 paint color clusters for each painting (excluding pixels belonging to the target). To this end, we computed the 3D boundary of each cluster (using the Matlab “boundary” function with a shrink factor of .8; The MathWorks, Inc.), tested whether a target color setting was inside one of these 3D volumes and counted the total number of “Innis” (target setting inside any one of the cluster-defined volumes in color space) per painting. We allowed for a tolerance distance ΔE*_ab_=15 at which a point would still be accepted belonging to a volume even if it was outside the boundary (for more information, see https://www.mathworks.com/matlabcentral/fileexchange/37856).

We also checked whether the *original* target color was inside the 3D color volume of the painting (simply put: Did the artist use a color that was already present in the painting), and whether observers’ color settings were the same as the *original* target color (remember, the 20 clusters above do not contain pixels belonging to the target region).

### Color Preference and Laterality Preference

To measure observers’ preferences for their own choices, we simply aggregated those cases per painting and compared these counts also between both observer groups. We followed the same approach for assessing the observer’s preferences for the original versus the flipped orientation. Since familiarity reduced the preferences for mirror-reversed images, we compared the orientation preferences between the 24 familiar and 24 additional unfamiliar paintings.

### Balance Point

We measured the size, as well as *x* and *y* location of the origin of observers’ balance points settings. To obtain a size estimate of the dot probe, we divided the diameter of the dot (in degrees visual angle) by the smaller dimension of the painting (in degrees visual angle). This scaling with respect to painting dimensions in the analysis was needed to truly capture the “importance” of the balance point: For example, a dot with a diameter of 5 in a square area with a side length of 6 cm has probably meant to have a different importance than a dot of the same diameter in a square with a side length of 20 cm. The distance of each the location setting (in degrees visual angle) was computed with respect to the geometric center (width/2, height/2) of a given painting. We compared dot probe size estimates and probe distances across 24 paintings and the two observer groups (Chinese and European).

We also investigated whether there was a relationship between laterality preference of familiar paintings (see earlier) and the perceived laterality of the balance point. To estimate the later, we first projected observers’ location settings onto the horizontal dimension of a given painting and then computed their mean distance from the origin. Values near 0 will indicate that balance points are perceived to be near the geometric center, that is, the horizontal midpoint of the painting, negative values indicate that balance points are perceived more on the left, and analogously, larger positive values indicate that they are perceived to be on the right side of the painting.

## Results

We will first present the results of the two color experiments and then the results of the two spatial experiments. For the *target color adjustment* experiment, we assessed first whether the distribution of participants’ color choices for the 24 targets were structured or random. After determining that they were mostly structured, we investigated in which way the color composition of the abstract paintings influenced the participants’ color choices for the respective targets.

### Minimum Distance Analysis and Target Color

**Figure 3.**
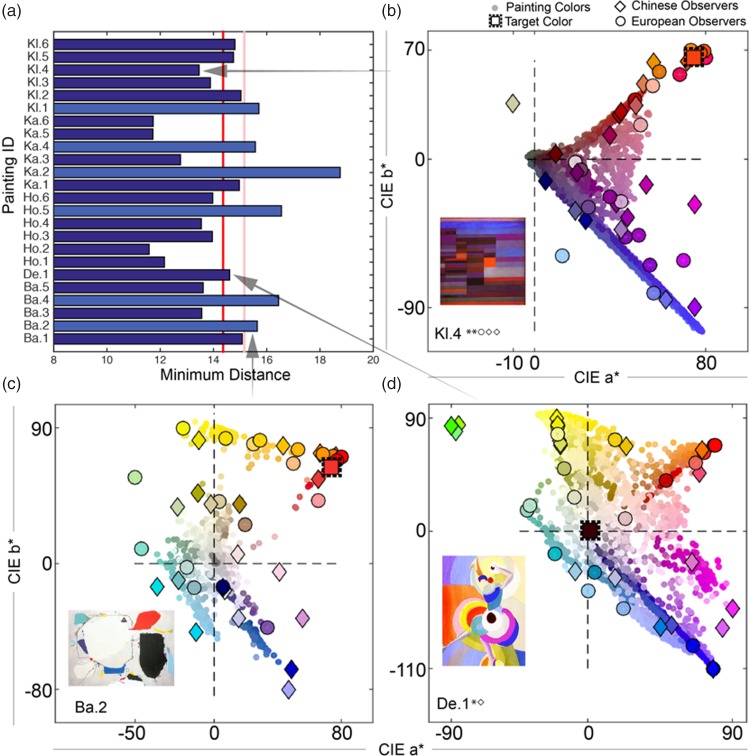
(a) Minimum distance analysis indicated structured target settings for 18 paintings. Bar length corresponds to the average minimum distance in CIE L*a* b* space between observer settings (Chinese and European participants combined). The red line corresponds to the 1% cutoff and the pink line to the 5% cutoff. Minimum distances that are larger than this cutoff value can be said to be yielded by random color settings (light blue bars). ((b) to (d)) Pixel distributions of paint colors of three paintings and corresponding observers´ target settings in CIE L*a*b* space. European participants are represented by circles and Chinese participants by diamonds. Star symbols indicate the significance of structuring in the participants’ target setting distributions as assessed by minimum distance analysis at the 5% (*) and 1% (**) cutoff level, respectively. Corresponding circle and diamond symbols indicate the significance of the amount of clustering for European and Chinese Participants, respectively. The original (mean) target color of each painting is represented by a square. Even though this representation of painting colors neglects the spatial distribution of paint colors, or the specific setting of color contrasts at the borders of elements, it illustrates nicely how the target settings were influenced by the painting colors—possibly more than what was revealed by the minimum distance analysis. Also see Supplementary Figure 2.

Figure 3(a) shows that for 12 paintings the minimum distances of participants’ target color settings were below the 1% cutoff (red line in Figure 2) and for 18 paintings below the 5% cutoff (pink line), indicating the participants’ color choices were (highly) structured. The distributions of target color settings for six paintings (Ba. 2, Ba. 4, Ho. 5, Ka. 2, Ka. 4, and Kl. 1 shown by lighter blue bars) were random. The European-Chinese group differences are presented in the Supplementary Figure 1. To give a more qualitative impression of the relationship between the distribution of colors of a painting and the distribution of observers’ target color settings, we plot target settings of European (circles) and Chinese (diamonds) observers together with the pixel distributions (small circles, no border, without pixels corresponding to target colors) of three sample paintings in CIE L*a*b* space (Figure 3(b) to (d)). Corresponding plots of all 24 paintings can be found in Supplementary Figure 2. Figure 3(b) shows an example of a painting (Kl. 4) that yielded highly structured target settings, overall and for both observer groups. Note that the target settings appear to be restricted by the distribution of paint colors. Conversely, the painting (Ba. 2) in Figure 3(c) has a much more “open” color composition. Even though our results indicated a random distribution of target settings, one can see that qualitatively this is not exactly true: The target settings are just substantially more spread out. We will explore the possibility that the variance of the painting colors influences the variance of the target settings below. Figure 3(d) shows an example of a painting that yielded a level of structure in target settings that lies somewhere between those of Figure 3(b) and (c). Even though the representation of painting colors in Figure 3(b) to (d) neglects the spatial distribution of paint colors, or the specific setting of color contrasts at the borders of elements, it nevertheless illustrates how the target settings were closely influenced by the painting colors.

#### The color of the target

Overall, participants tended to choose more yellow, red, and orange hues for the targets, which—interestingly—corresponded quite well to the distribution of the original target colors of the paintings, while bluish colors were selected much less (see Supplementary Figures 3 and 4), although the color blue is known to be the most favorite color of humans (Choungourian, 1968; Ou, Luo, Woodcock, & Wright, 2004; Palmer & Schloss, 2010; Saito, 1996; Schloss & Palmer, 2011; Taylor et al., 2013). In our exiting survey (Supplementary Table 3), blue tended to be a popular color, as were red and green. Yet, favorite colors distributed quite differently than the target settings: Among Chinese participants, shades of red, blue, yellow, and purple were named with equal frequency as favorite color (each 20%), followed by orange (10%); green and shades of cyan were never among the favorites. Among European participants, shades of red and green were named as favorite color (each 30%), followed by blue and yellow (15%) and purple (10%); orange and shades of cyan were never among the favorites. Thus, color preference cannot account for the overall target setting distribution.

### Influence of painting hue variance on target hue variance

**Figure 4.**
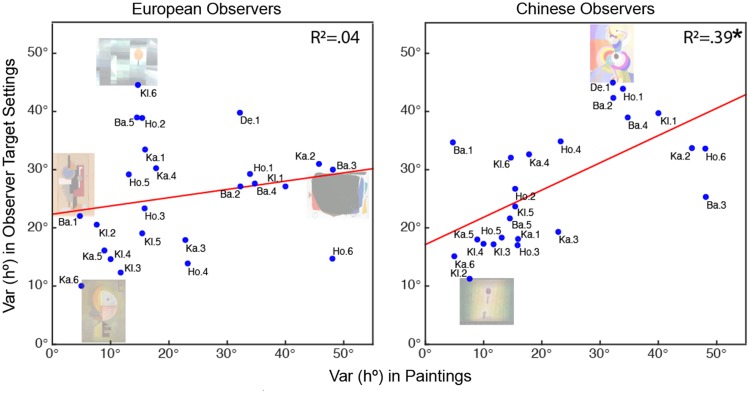
Influence of hue angle variance of the painting onto hue angle variance in observer target settings for both observer groups. The star symbol denotes significant correlations. Only for Chinese observers, we found a significant correlation between painting hue angle variance and their color settings for the targets.

We find that hue angle variance in paintings systematically influenced the hue angle variance for overall target color settings (*R*^2^ = .28, *p* < .007). At the individual group level, this relationship was true for Chinese (*R*^2^ = .39, *p* < .001) but not for European observers (*R*^2^=.04, *p* = .33). Figure 4 also illustrates that a smaller hue angle variance, as in the Baumeister painting (Ba. 1) or Klee’s painting (Kl. 6), does not necessarily produce a small range of target color choices. Variance in luminance and chroma values in the paintings did not systematically influence the variance in either observer group’s target settings (Luminance: *R*^2^_Ch_ = .034 and *R*^2^_Eu_ = .06; Chroma: *R*^2^_Ch_ = .005 and *R*^2^_Eu_ = .07).

#### Luminance and chroma correlations

**Figure 5.**
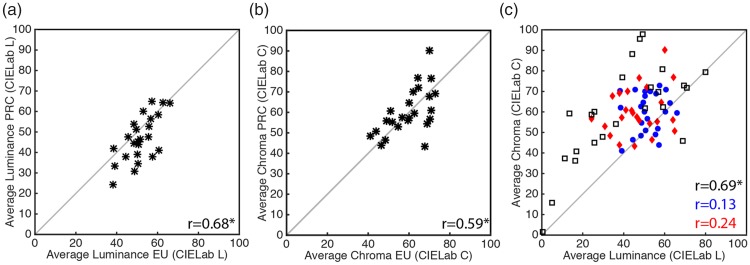
Luminance and chroma correlations for target settings. Overall, the luminance and chroma target settings of the two observer groups correlated significantly. While there was a high correlation between CIE L* and C*ab values of original target colors, there was no significant correlation between those variables for either observer group. Also see Supplementary Figure 5 for average luminance and chroma settings for each observer group and painting.

Overall, the target luminance (Figure 5(a),*r* = .68, *p* < .0002) and chroma settings (Figure 5(b),*r* = .59, *p* < .0024) of the two observer groups correlated significantly. However, there was no significant correlation between luminance and chroma values for either observer group (Figure 5(c), red and blue symbols). For original target colors, however, there was a high correlation between those two variables (Figure 5(c), black squares). Interestingly, Chinese observers tended to make their targets darker than European observers (CIE L*Ch = 46.75 and CIE L*Eu = 51.66; also see Supplementary Figure 5).

### Agreement of painting color space and target settings

**Figure 6.**
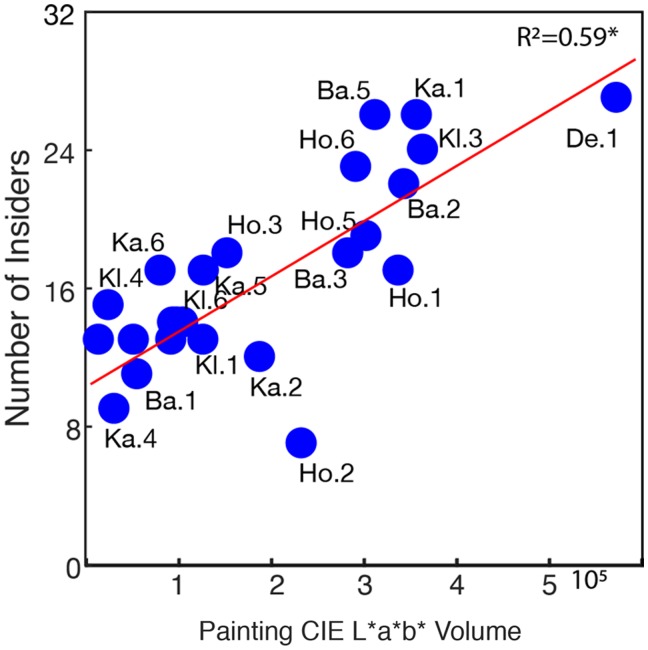
Relationship between CIE L*a*b* painting volume and number of insiders. CIE L*a*b* painting volume (size) predicted the number of insiders. We omitted the painting labels for overlapping data points in order to avoid visual clutter in the graphs. Supplementary Figure 6 shows the proportion of target color settings inside CIE L*a*b* painting space as well as separate regression analyses for European and Chinese observer groups.

**Figure 7.**
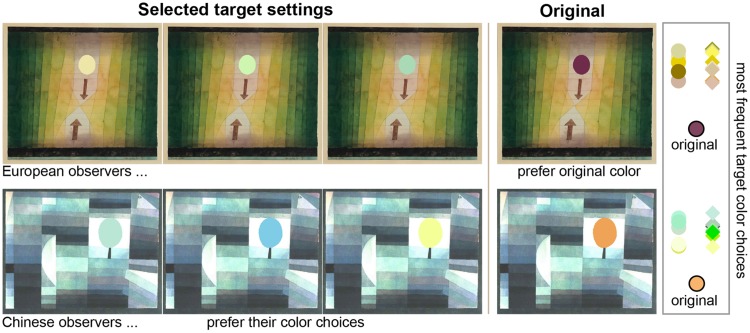
Examples target settings and different preferences of both observer groups. Even though the target settings of both observer groups could be quite similar, their preference for the target color could differ (on the right: round symbols denote European target settings and square symbols Chinese target settings). Most European observers preferred the artist’s target color over their own choices for Kl. 2, and liked equally their own target color choice for Kl. 6, while most Chinese observers preferred their color for Kl. 6 and equally liked the artist color for Kl. 2. Interestingly, observers tended to choose colors that contrasted in lightness with the average painting color. Schloss and Palmer (2011) found a similar tendency studying color preferences for pairs of uniform color patches, also see Supplementary Figure 5.

Both observer groups tended to choose target colors that were also contained in the color palette of the painting, that is, observers’ target color choices were inside the volume (“insiders”) delineated by the 20 CIE L*a*b* clusters that each painting was segmented into. Target color choices, which remain inside the color palette of the painting, support the “harmony” of the color composition. In addition, for half of the paintings, the artist adopted this strategy (bold printed painting IDs in Supplementary Figure 6, Panel A). Figure 6 illustrates that the larger the volume in CIE L*a*b* space the more frequently observers tended to pick a target inside the painting space (also see Supplementary Figure 6). If observers adopted a strategy where they randomly selected colors from the painting distribution volume in CIE L*a*b*, larger color volumes would predict more variability in the selected target colors. A regression analysis somewhat supports this idea (*R*^2^=.182, *p* = .038); however, the prediction is quite weak. Therefore, it is likely that target color hues might have been selected according to additional criteria, such as picking a color that is similar in hue but contrasts in lightness with adjacent regions (as in Kl. 4, also see Figure 7).

Painting hue variance did not predict the number of *insiders* (European observers: *R*^2^=.024, *p* = .467; Chinese observers: *R*^2^=.007, *p* = .684). Note that painting hue variance and painting CIE L*a*b*Color volume were also not correlated (*r* = .364, *p* = .082), which means, for example, that for a given painting, a larger hue variance in a painting not necessarily translated into a larger volume in CIE L*a*b*.

We also wanted to know whether the overall color composition of a painting would “force” observers to make a color choice similar to the artist. Put differently: Is there only one way to complete this painting? We found that only 2.1% of Chinese observers’ and 1.7% of European observers’ target settings were inside the volume consisting of the 5 CIE L*a*b* clusters of the original target color, which means that only few observers arrived at exactly the same solution as the artist (Supplementary Figure 6(a), square symbols, but also see Supplementary Figure 4).

### Target Color Preference – “Kandinsky or me?”

At the end of the entire experimental session, we wanted to know whether participants would prefer their own color settings for the targets when they would see their version of the painting next to the original. As a reminder, to allow for a fair comparison between both image versions, we also replaced the target region in the original painting with its average color. Overall, observers seemed not to have strong preferences over their own or the artists target color choice. Figure 7 shows an example, where the European observers preferred the artist’s target color over their own choices and one, where the Chinese observers tended to prefer their own target color choice. Even though the target settings of both observer groups could be quite similar, their preference for the target color could differ. Supplementary Figure 7 plots the proportion that the artist’s target color was preferred for each observer group and each painting.

### Position of the balance point in a painting

In this experiment, participants had to indicate the position and strength (importance) of the “balance point” or “center of gravity” for each painting by adjusting the location of a black filled circle in an adjacent white rectangle, which had the same size and proportions as the corresponding painting. Overall, observers were able to do this task, choosing the “no balance point” option only on 17.4% of the trials (Supplementary Figure 8, red and blue bars). Settings for circle size, which indicated the strength of the balance point, varied significantly across paintings and observer groups (Supplementary Figure 8 dot and diamond symbols). However, the size of the dot probe differed significantly in size for only a few paintings: for example, for Ba. 3, it was significantly larger than for all other paintings, and for Kl. 2 and Kl. 3, dot probe sizes were smallest.

**Figure 8.**
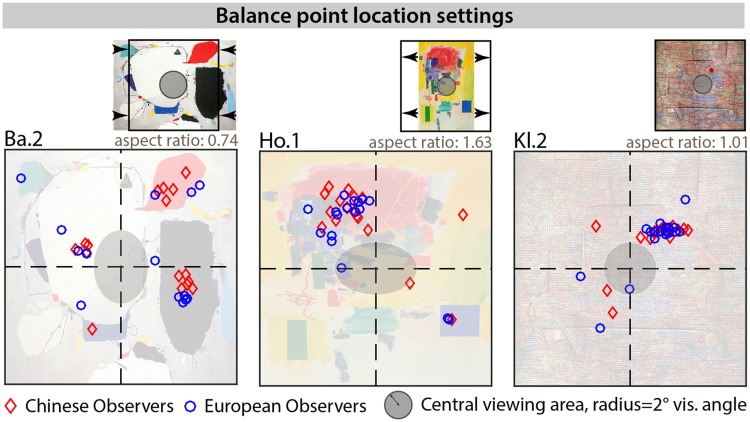
Selected balance point settings for three sample paintings and *x* and *y* coordinates of the circle probe setting for each observer (Chinese: red diamonds, European: blue circles). All images and data were scaled to fit the panel dimension, original aspect ratios (height/width) are given; black dashed lines bisect each painting horizontally and vertically. The central gray circle (also scaled along with the painting) has a radius of 2 degrees visual angle and approximately corresponds to the central region of each painting. In this representation, a painting that is larger in height than in width will look compressed along the vertical dimension (e.g., Ho. 1) and the central gray area will be shaped like a horizontal ellipsoid. Also remember that in the experiments, all paintings were scaled along their largest extend (width or height) to approximately 20 degrees visual angle. This figure illustrates that, in general, observers use the painting structure to estimate the balance point. Supplementary Figure 9 shows balance point settings for all paintings.

The estimated locations of the balance point are plotted for each painting and observer group (Chinese: red diamond symbols, European: blue circles) are shown for three examples in Figure 8 and for all paintings in Supplementary Figure 9. Overall, there was quite good agreement between both observer groups and their balance point settings for the different compositions of the 24 paintings. A 2 (observer groups) × 24 (paintings) analysis of variance (ANOVA) on the distance between setting and painting’s geometric center supports this observation yielding a main effect of painting ID *F*(1,23) = 10.15, *p* < .0001, and no main effect of observer group. There was also a significant interaction *F*(1,23) = 1.9, *p* < .0067, suggesting that for some paintings, the distance of the balance point to the center of the painting was different between observer groups. However, following up the interaction yielded no significant differences between observer groups for any of the paintings. On average, the balance point distance to the center was about 2.1 degrees of visual angle for both observer groups, suggesting that observers used the content and structures of the painting for their setting and tended to not choose the “trivial solution,” that is, the geometric center of the stimulus (intersection of the black dashed lines in Figure 8 and Supplementary Figure 9). In Figure 8, the central gray disk demarcates an area within each painting of about 4 degrees visual angle diameter. For most paintings, settings for the balance points tended to be outside this central region, indicating that their structures were not perceived as symmetrical.

### Left-right orientation of paintings

In the third of four experiments, participants had to indicate whether preferred the original or the mirror-reversed version of 24 familiar (used in the previous experiments) and 24 unfamiliar (though painted by the same artists) paintings. Supplementary Figure 10 illustrates that preferences for mirror-reversed paintings were significantly lower for the 24 familiar paintings, and a 2 (groups) × 2 (familiarity) ANOVA on laterality preferences confirms this, yielding a main effect of familiarity *F*(1,92) = 43.35, *p* < .0001. There was no significant difference between Chinese and European participants in terms of their laterality preference.

It is well known that familiar paintings are preferred in their original orientation (Gordon & Gardner, 1974; Lindauer, 1969; Mather, 2012; B. M. Ross, 1966; Swartz & Hewitt, 1970). Here, we were interested to find out how the perceived spatial composition of a painting might influence the orientation preference. Particularly, we wondered whether the location of the balance point settings of our participants might be correlated with their preferences for the original or mirror inverted orientation of a painting. For example, paintings with a perceived balance point off to one side may be more likely to be accepted in their original orientation than those with a more symmetrical composition and a perceived balance point close to the geometric center of the painting. To test this hypothesis, we plotted the orientation preference of our participants as a function of the laterality for the perceived balance point (center of gravity), that is, the average distance of the *x*-value of the balance point with respect to the horizontal midpoint of the painting. Negative values defined positions of the origin on the left side of the horizontal midpoint; positive values defined positions on the right side. We combined the data of both groups for this analysis, since there were no differences in balance point location and laterality preference between Chinese and Europeans.

**Figure 9.**
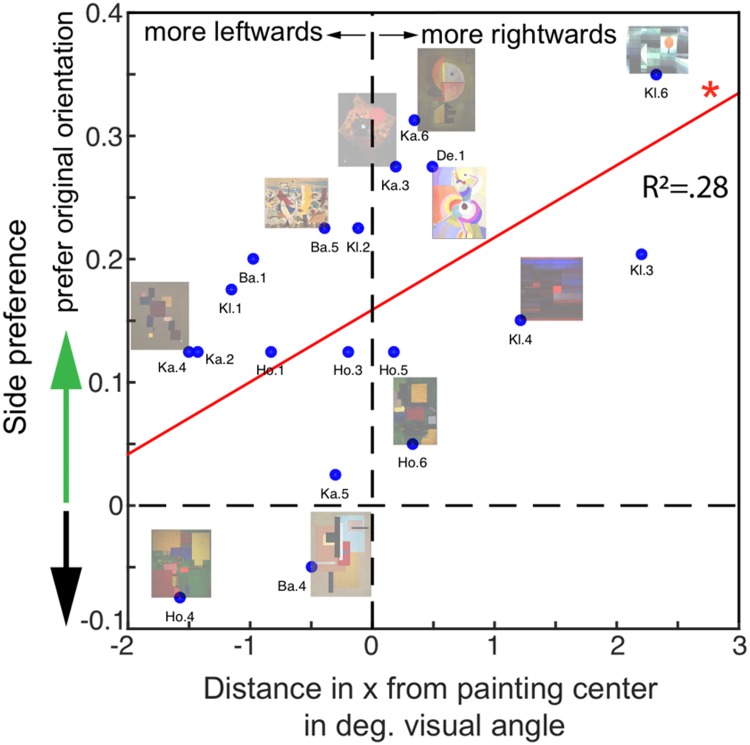
Spatial relationship between the perceived center of gravity in the original painting and the preference for orientation. Along the *x*-axis, we plot the average distance (in degrees of visual angle) of the origin of the circle with respect to the horizontal midpoint of the painting. Negative values specify a leftward distance of the circle probe to the center; positive values specify a rightward distance of the dot probe to the center. On the *y*-axis, the proportion of the average side preference of the observers is plotted. Positive values indicate that the original orientation was preferred, negative values that the flipped version was preferred, and values close to 0 imply no preference. The relationship between side preference and dot distance was significant: The more a balance point was perceived on the right side, the more the original orientation of the painting was preferred; the more leftward the balance point was perceived, the more observers preferred the flipped version. The shift of the data toward “prefer original orientation” suggests a memory effect. Note that regressing side preferences onto relative *x* displacements, that is, dividing the distance in *x* from the painting center by the size of *x* dimension of the painting, does not change this relationship, if anything, it becomes slightly stronger (*R*^2^ = .29, *p* = .02, see Supplementary Figure 11).

We first computed orientation preference as an index between –.5 and .5, where 0 corresponds to no preference, negative values correspond to flipped orientation preferred and positive values to original orientation preferred. The laterality of the balance point was computed by computing the mean distance of the origin of the settings projected onto the horizontal dimension. Values near 0 indicated that balance points were near the geometric center, the horizontal midpoint of the painting, while larger negative values indicated that balance points were perceived more on the left, analogously, larger positive values indicated their perception on the right side of the painting. Our prediction was that orientation preferences near 0 would correspond to laterality indices near 0, and conversely, nonzero orientation preferences to nonzero laterality indices. We also reasoned that there are three ways to obtain a spatial mean of 0: (a) if settings were truly near the painting’s center; (b) if settings were highly dispersed, or (c) if settings clustered equally on left and right sides of the painting, indicating two or more centers of gravity. Only the first case would indicate a truly centrally perceived balance point. To exclude the other two cases, we excluded six paintings (Ba. 2, Ba. 3, Ho. 2, Ka. 1, Kl. 1, and Kl. 5) for which the variance in the balance point setting (horizontal dimension) exceeded 20 degrees of visual angle (20 degrees was the maximum height or width a painting could have, thus settings must have been pretty spread out to achieve this kind of variance). Please note that the results do not change if we would leave the data for these six paintings in the analysis. In Figure 9, the relationship between the perceived location of the balance point (center of gravity) and orientation preference is plotted. The positive shift of the regression line illustrates the expected overall effect of memory, that is, our observers tended to prefer the 24 familiar paintings in their original orientation. The positive slope of the regression line suggests that the more rightward the balance point is perceived, the more the original orientation of the painting is preferred. Figure 9 demonstrates that there is indeed a significant relationship between these two indices (*R*^2^ = .28, *p* < .021). In essence, for our set of paintings, the likelihood that the mirror reversed orientation of a painting is preferred increases with the left sidedness of the perceived location of the balance point. This latter is a surprising result, which we discuss later.

## Discussion

### Summary

Abstract art offers a unique possibility to study aesthetic judgments, the sensitivity to color composition, spatial structure, or compositional weight with little influence of top-down factors (Hurlbert, 1996; Palmer et al., 2013; Tanaka et al., 2001; Tanaka & Presnell, 1999). Using an interactive task, we found that colors of abstract paintings systematically influence art naïve participants when choosing a color for a single painting element (target). Specifically, the hue variance of painting colors influenced the hue variance of target settings, and larger volumes formed by painting colors in CIE L*a* b* are more likely to make observers choose an existing hue already used in the painting for their target (rather than a novel one). Almost never observers chose exactly the same colors for the target elements as the artists; however, they often tended to select a hue in the same category. Accordingly, they liked most of their own solutions as much as the artists. We also investigated the perception of the compositional structure of the paintings and discovered a remarkable consistency between the balance point settings and the laterality preferences of all observers. The more the balance point was perceived on the right side of the painting, the more observers preferred the original orientation; the more the balance point was perceived on the left, the more the mirror-reversed version was preferred. The comparison of the results of our Chinese and German student groups revealed differences in target color luminance settings, in the strength of the painting’s hue variance and in the preferences of color settings for individual paintings.

### Influences on target color selection

#### Hue variance, color volume, and contrast

In our interactive color experiment, the target color was selected against a multicolored background, that is, the painting. The whole color composition of a painting systematically influences observers when they search and select a “suitable” color of a single element presented within the artwork. While individual observers can arrive at very different “solutions” for a given painting as the artist—at a group level (considering all observers)—higher hue variance in the artwork leads to higher variance in the target colors chosen by observers. However, this is a rather modest effect. Hue angle variance does not predict which target colors participants will choose. What appears to influence the latter is the size of the color volume of the painting, that is, the space that colors of a painting take up in CIE L*a*b*. The larger this color volume of the painting is, the more often observers pick a target color that is already present in the painting. Conversely, smaller color volumes cause observers to venture out of the painting’s color palette for their target setting. One might argue that any larger volume would “capture” more of the observers’ target settings—even if these were completely randomly distributed in CIE L*a*b*. However, we can exclude randomness in the color settings for nearly all (18 of 24) paintings, as shown by the minimum distance analysis. Thus, it appears that having many different shades of colors in a painting “entices” participant tend to select a target color that is already present in the painting. Why might this be? Using a simple color pair stimulus, Schloss and Palmer (2011) also found that observers’ preferences for color combinations were dominated by hue similarity (rather than by preference for the individual colors). This preference for hue similarity seems to generalize to more complex stimuli, such as those used in the present study.

A rich set of paint color provides lots of examples for the specific color composition of an artwork. When only a few colors are present, the participant may want to increase color variance with his/her setting, because repeating a color when only few colors are present might lead to an imbalance, or perhaps there is an optimum number of colors or contrasts that the observer is implicitly striving for.

The relationship between complexity and aesthetics has been considered before. For example, according to Birkhoff (1933), *aesthetic measure* (*M*) is always equal to *the number of elements of order* (*O*) divided by the *complexity* (*C*). In his equation: *M* = *O*/*C*, the aesthetic measure is determined by the relation between the elements of order and complexity. A high aesthetic measures can be achieved by a very simple design (a low value of *C*) or by an orderly arrangement of elements (high value of *O*). Birkhoff applied his measure to ornaments, shapes of vases, musical harmony and melody, and poetry, but not to color and paintings. An application of this equation to color compositions in a painting requires the determination of the weights to be given to the elements of order and complexity. For color he gave the advice:

“The simpler the palette is, the less will be the complexity, so that the palette should be as restricted as the subject permits. Evidently the eye appreciates the repetition of a color, a graded sequence of colors, and a balance of light and dark values about the centers of interest, …” (p. 213).

It appears, that not only art-receivers (i.e., our participants) but also artists tend quite often to prefer target colors that are already present in the painting—perhaps to support the “harmony” of the color composition (Moon & Spencer, 1944; Ostwald, 1917; Ou, 2015).

We did not explicitly ask observers to make harmonious color choices, but color harmony might still have influenced their color preferences. In fact, the terms color preference and harmony were often used interchangeably (Chevreul, 1839/1967; Granger, 1952). Moreover, in the past *color harmony* has been studied mainly with simple stimuli (Helson & Lansford, 1970; Itten, 1973; Munsell, 1912; Nemcsics, 2007; Ostwald, 1917; Ou et al., 2004), and it is thus not well defined for complex compositions like abstract paintings. Schloss and Palmer (2011) explicitly tried to distinguish between aesthetic preferences for color combinations and perceived color harmony by asking observers to indicate first, whether they liked (preference) a given color combinations of a smaller square on a larger one, and then to rate the harmony of the same color pairs. To measure configural influences on preference, observers also had to evaluate the combination of a figural color against a uniform colored background. The researchers found that more similar hues were preferred more and also rated as more harmonious, consistent with Chevreul’s claim (1839/1967). Interestingly, when a color was rated as a figure color on a background, preference ratings increased as the hue contrast between figure and background increased, similar to findings by Helson and Lansford (1970). We find both tendencies in our data, that is, color preferences (Experiment 4) that either support color contrast or harmony. Interesting are the two paintings, where most of our observers disagreed with the artists and chose rather harmonious colors: For his painting Ba. 4, Baumeister used a light blue on a red rectangle, while 43% of our observers chose a yellowish hue. In the painting Kl. 6, Klee used orange for the target element to indicate autumn on a white background surrounded by different shades of light green, blue, and gray. Here observers selected mostly greenish hues (38%).

One final point to consider is that we used JPEG images of the paintings to present them on a monitor and that reproduction errors might have substantially distorted the overall color harmony of the original painting and thus affected observer’s target color choices. In fact, it is possible that the original painting might have yielded a different or less variable target color settings. How reproduction errors influence perceived colors in paintings may be investigated in future studies.

#### Shape context

The choice of a color for a single element may be influenced by its shape as suggested by Kandinsky (1912, 1947). He described interactions between shapes and color, that is, the strength of a certain color was either supported or weakened by a certain shape. His assignments of shapes to colors, that is, squares with red, triangles with yellow, and circles with blue were based on his own associations with colors, that is, like “sharp” for yellow and “dense” for blue (Kandinsky, 1912, pp. 101–102). In a recent study about the association of hue and shape by Albertazzi et al. (2013), color choices for shapes were not random and the strongest associations were found for triangles and yellow and circles and squares and red. In our study, most targets elements were rectangles, only seven were circles, and one was a triangle and their sizes differed. Since our observers preferred in general reddish and yellowish hues and neither the number nor the size of the shapes were adjusted, it was not possible find any consistent shape/color associations as suggested by Kandinsky (see Figure 1(a) and (b) and Supplementary Figure 4). Even for his own paintings (Ka. 1 to Ka. 6), Kandinsky chose a variety of hues for rectangles and circles; this was also true for the other artists. Another factor may be the relative size between the target and the areas of juxtaposed colors and the surround. Here an important element of color harmony is the spatial balance of the color components (Albers, 2013; Morriss & Dunlap, 1987). Koenderink et al. (2018) found strong interindividual differences when observers’ judged compositional weight based on luminance.

In contrast to our initial idea, we found that the surrounding context of a painting rarely predicted the *exact* target color. However, when relaxing the criteria of “sameness” a little, many observers tended to stay in the same (or a similar) hue category as the artist (see Supplementary Figure 4). Thus, the perceived hue of the target was often influenced by the multicolored surrounding of the corresponding painting. It is well known that the perception of color depends not only on its surface reflectance (and its illumination) but also on the colors of the surround (Bloj, Kersten, & Hurlbert, 1999; Brainard & Maloney, 2004; Brainard & Wandell, 1992; R. O. Brown & MacLeod, 1997; Foster, 2011; Hurlbert, 1996; Shevell & Kingdom, 2008), a fact also painters are well aware of (Albers, 2013; Mollon, 2006). Color interacts with the surrounding regions, for example, stimuli seem to assume the brightness and color of the surround, as in the perceptual phenomenon of filling-in (Friedman, Zhou, & von der Heydt, 1999; Krauskopf, 1963; Paradiso & Nakayama, 1991), or the long-distance inductions of color in the so-called *watercolor effect* (Katz, 1911; Pinna, 2012; Pinna, Brelstaff, & Spillmann, 2001). During the color adjustment experiment, our participants experienced continuously color contrast effects of the adjacent surround. They often “tried out” target colors by going back and forth between certain hues and spent quite some time to settle on a final decision. Just as for artists, the observers’ final color choices were the result of many decisions, based on the complex interactions between the target color and the simultaneous color contrast of lines, borders, and shapes (Brainard, Wandell, & Chichilnisky, 1993; R. O. Brown & MacLeod, 1997; Shevell & Kingdom, 2008; Singer & D’Zmura, 1994).

#### Kandinsky or me!

Overall, participants were as likely to prefer “their version” of the target as the original. This might be because they frequently picked target hues in the same category as the artist, but it could also imply that the color of a single element did not exert an influence on the overall aesthetic experience of the painting—though artists would definitely disagree with this interpretation! In contrast, rotating the entire color gamut of paintings, Nascimento et al. (2017) found that art-naïve observers (and art specialists) strongly preferred a chromatic composition very close to the original gamut orientation. This stronger preference for the original gamut orientation can be explained by the more dramatic effects of rotation, which affected all hues of a painting (Nascimento et al., 2017).

In our experiment, the effect of color was limited to a single element and their sizes differed by large amounts. One of our initial questions was whether participant would choose a similar color as the artist for an element to “finish” a painting. The fact that participants’ target settings were not random but often chose a hue of a *similar category* as the artists’ original target color, suggests that for some paintings the larger color context of a painting may indeed “limit” the possible hues for an element of a given color composition, and our art-naive observers appear to be sensitive to this. The multicolored surrounding of paintings often influenced the final color choice. Even for the smallest target of the image Kl. 3, 75% of the European and 50% of the Chinese observers picked hues from the category red and more than 60% preferred the artist’s or their choice of the color red.

### Compositional weight and left-right orientation

In the second interactive task, we found that participants agreed substantially in their balance point settings and tended to use “landmarks” within the painting *instead of geometrical measures* like the geometric center of the image. This suggests that our task balance point was feasible and intuitive. Many factors influence the subjective impression of compositional weight or perceptual balance, including the geometry of the canvas; the distribution, orientation, and size of different elements; and the placement of colors and color contrast (Bertamini, Makin, & Pecchinenda, 2013; Graves, 1951; Morriss & Dunlap, 1987; Moon & Spencer, 1944; Palmer et al., 2013; Pinkerton & Humphrey, 1974; Wong, 1993; Wright, 1962), orientation (Fuchs, Ansorge, Redies, & Leder, 2011), and luminance contrast (Itti & Koch, 2000; Koenderink et al., 2018). In addition, in our study, properties of the target shape might have influenced the perceived balance, for example, the relative size of the balance point seemed to be influenced by the general sizes of the elements present in each painting as in Ba. 3, which contained a dominant large black element, or in Kl. 3, which consisted of tiny colored spots and lines. Yet, overall, participants did not use the radius setting much. This may have several reasons: the translation of strength (salience of the balance point) into the size of the radius was too unnatural, the paintings were perceived as being generally balanced, or more than one center was present and the radius of the circle was set to the relative strength. Our results are consistent with previous studies which showed that observers are quite sensitive to the relative position of objects of different length, color, and size and their spatial relationships within a frame (Arnheim, 1954/1974; Bertamini, Makin, & Rampone, 2013; Fechner, 1876; Leyssen, Linsen, Sammartino, & Palmer, 2012; McManus, Edmondson, & Rodger, 1985; McManus, Stöver, & Kim, 2011; D. W. Ross, 1907; Wilson & Chatterjee, 2005).

#### Laterality

Whether the original orientation of a painting or a photograph is important for the whole impression has been investigated by rotating or mirror-reversing an artwork (Mather, 2012; Swartz & Hewitt, 1970). Having seen the paintings during the first two experiments shifted preference of our participants toward the original image orientation. This memory effect on laterality judgments has been well documented in the literature (Mather, 2012; Shepard, 1967; Standing, Conezio, & Haber, 1970). What is more surprising is that the more leftward the participants perceived the location of the balance point in the original painting, the more they preferred the mirrored version, while the more rightward they perceived the balance points, the more they preferred the original orientation. Why might this be?

The preference of a certain orientation of a painting or photograph depends of course on the subject, that is, portraits or landscapes are rarely preferred upside down, because recognition for faces is better with the up-right orientation but also the perception of depth (Bartley & Dehardt, 1960) and illumination are important. For ecological reasons, humans have a preference for a light source position above their heads and therefore prefer paintings lit from the top but also slightly to the left (Gombrich, 1961; Mamassian, 2008; Mamassian & Goutcher, 2001; McManus, Buckman, & Woolley, 2004; Metzger, 1936). As, in abstract art, figurative objects, which would require a certain orientation, are rare or absent, these types of artwork could in principle be hung any way, but often just one orientation seems to work best. The distribution of luminance may be one, because mostly lighter areas are preferred at the top and darker ones at the bottom (Gombrich, 1961; Mamassian & Goutcher, 2001; McManus et al., 2004; Metzger, 1936). Other reasons are the perception of depth (Bartley & Dehardt, 1960), the balance or relative heaviness of elements and the movement direction of the scanning process (Braine, 1968; Brandt, 1940; Buswell, 1935; Gaffron, 1950), or the presentation time (Freimuth & Wapner, 1979). Moreover, artists turn their paintings to look for the overall balance of their artwork from a different perspective. For abstract art, the preferred orientation may therefore change during the process of painting.

The preference for rightward balance points agrees with the suggestion of Wölfflin (1928) that observers have the tendency to scan a picture from left to right and that scanning ends on the right picture half, where the most important content is presented. This would explain why the preferences for the mirror reversed images were very low when the original had its balance point on the right side, because scanning of the most important content of reversed version of the images would take place at the beginning, that is, on the left side. The origin of the rightward balance point bias might have its roots in several aspects: One might be handedness (Levy, 1976) or reading habits (Chokron & De Agostini, 2000; Ishii, Okubo, Nicholls, & Imai, 2011). All of our participants were right handed and read from left to right. This preference for balance points located on the right might also explain results of studies (e.g., Gordon & Gardner, 1974; Lindauer, 1969; B. M. Ross, 1966; Swartz & Hewitt, 1970) which reported that observers were at chance to detect (or prefer) the correct orientation of paintings. It could be that the perceived balance point of the artwork used in these studies tended to be more central, for example, in art works by Pollock, Davis, or DeKooning used by Lindauer (1969).

### Cultural differences

Our sample of 20 Chinese and 20 German participants is certainly too small to generalize our findings to a larger scale of cultural differences in color preference or perception of compositional weight. However, our two groups were relatively homogenous; both consisted of art naïve students of similar age, gender, academic education, and social background. In fact, our exit poll at the end of the experiments indicated that interest and preferences in art, favorite artists and colors, and liking of the stimuli were rather similar for both groups: Most of the participants did not know even any of the painters, the best known painter was Kandinsky and abstract art was usually not the preferred art style. Therefore, we believe that the observed differences between the results of both groups in our two color experiments may be primarily due to the overall cultural differences (Beke et al., 2008; Hurlbert & Owen, 2015; Park & Guerin, 2002; Park & Huang, 2010; Silva, 2006; Yokosawa et al., 2010, 2016).

Interestingly, all differences that we find between our two cultural groups—with the exception of the Chinese choosing slightly larger sized balance points—emerged with respect to color. Chinese observers preferred slightly darker target colors and disliked strong color contrasts as in Kl. 3. In traditional Chinese paintings often relatively few colors are used for the representation of objects in the foreground, such as blossoms, animals, or human clothes, while the surround consists of darker structures or areas, such as landscapes, branches, or buildings on relatively light homogeneous background (Barnhart, Xin, & Chongzheng, 1997; Li & Gardner, 1993). This way the few colors are salient and the focus of the painting is immediately clear. Overall, Chinese observers proved to be more “sensitive” to the original color composition of the art work, their hue variance for target color settings was more strongly influenced by the variance of the hue angle in paintings, and the relationship between painting color volume and the inside-target settings was stronger. Sensitivity to color harmony (Moon & Spencer, 1944; O’Connor, 2010; Palmer et al., 2013) has been suggested to be determined not only by the individual characteristics of the observer such as his/her age, gender, personality, and affective state but also by cultural experiences (Leder et al., 2004; Yokosawa et al., 2016).

### Intuitiveness of the tasks

How visual art is perceived, which factors influence the process of aesthetic appreciation, is a newly emerging area of research (Brown, Gao, Tisdelle, Eickhoff, & Liotti, 2011; Chatterjee & Vartanian, 2014; Cinzia & Vittorio, 2009; Conway, 2012; Gartus, Klemer, & Leder, 2015; Ishizu & Zeki, 2011, 2013; Leder et al., 2004; Leder, Gerger, Dressler, & Schabmann, 2012; Silva, 2006; Vartanian & Skov, 2014; Zeki & Nash, 1999), although already in the late 19th century Fechner (1876) and Wundt (1896) began to investigate visual properties of artworks and introduced aesthetic experience of art as a discipline of visual perception (also see Lange, 1901). As a new field, it is still struggling with honing in on the best methods and technologies. While many studies have focused on measuring explicit statements of “liking” or beauty (Brielmann & Pelli, 2017, 2018; Fechner, 1876; Leder & Nadal, 2014), we introduced here an interactive approach to study how observers perceive the color and spatial composition of abstract artworks (for other examples of interactive tasks, see also Koenderink, van Doorn, & Wagemans, 2017; Nascimento et al., 2017).

All our observers were art-naïve; most of them did not know any of the artists or had seen any of the paintings before. However, to choose the “paint” for an element within an abstract painting, and to experience the effects single colors can have within complex color compositions, caught the interest of our observers immediately. To navigate in color space and to try to find a “good” color turned out to be a quite intuitive task (Koenderink, van Doorn, & Ekroll, 2016). To “play” with color within a colorful surround like in our experiment seems to be a very pleasant experience, as Conway (2012) writes: “we like color” and color “appears to have a direct impact on the limbic system” (for a review of color emotion and color harmony, see Ou, 2015; Solli & Lenz, 2011). Even though our observers were not interested in art, especially in abstract art (Supplementary Table 1), they stated that they enjoyed our interactive color adjustment task although they realized the difficulty to find the “right” color for some of the paintings. Our interactive tasks were very open-ended and some of the variability between our participants might be explained by idiosyncratic differences in how individuals understood the instructions and what criteria they applied in doing the task. Overall, we find quite good agreement in our data for all tasks, not only between individual participants but even across the two cultural groups.

## Conclusions

Abstract art offers a unique possibility to study aesthetic judgments. Using two novel interactive tasks, we found that art naïve participants show high sensitivity to the color composition (hue variance and color volume) of abstract paintings and often arrive at similar “solutions”—when they had the task to find a suitable color for a single element in different paintings. When we investigated the perception of the compositional structure of the paintings, we discovered a remarkable consistency between the balance point settings and the laterality preferences of all observers. The more the balance point was perceived on the right side of the painting, the more observers preferred the original orientation; the more the balance point was perceived on the left, the more the mirror-reversed version was preferred. Cultural factors appear to influence the sensitivity to color composition for young art-naïve observers.

## Supplemental Material

Supplemental material for Kandinsky or Me? How Free Is the Eye of the Beholder in Abstract Art? Click here for additional data file. Supplemental Material for Kandinsky or Me? How Free Is the Eye of the Beholder in Abstract Art? by Doris I. Braun Katja Doerschner in i-Perception
