# Supplementary material for: Kandinsky or Me? How Free Is the Eye of the Beholder in Abstract Art?
Source: Iperception. 2019 Sep 4;10(5):2041669519867973. doi: 10.1177/2041669519867973 (PMC6755862; doi:10.1177/2041669519867973)

Supplementary Text 1

Exiting survey.

1. Are you interested in art: yes very much/ yes/ it depends / not really / no
2. How often do you go to an art exhibition or art museum?
3. What style of art do you personally like best?
4. Do you like ‚Abstract art'?
5. How many paintings of the third experiment (side) do you know?
6. Do you know any of the artists?
7. Which task did you like?
8. What is your favorite color?

Supplementary Table 1. **Artists, titles and dates of the 24 paintings used in all experiments. We selected paintings of 5 artists who were painted during approximately the same time period, roughly the first half of the 20th century. Wassily Kandinsky (1866-1944) studied law and economics, before he gave up his career to learn art at the age of 30. Kandinsky's creativity is not only present in his artworks painted in very different styles but also in his theoretical work about form and color. Paul Klee (1879-1940) studied art in Munich and focused at first on graphic art. After his trip to Tunis he painted his first abstract painting 1914. Later he became a member of the 'Blue Four', a group founded 1923 together with Kandinsky, Feininger and Jawlensky. Willi Baumeister (1889-1955) studied art in Stuttgart and worked as professor in Frankfurt, until he was dismissed by the National Socialist regime. With his multiform and –colored paintings he left the traditional connection between color and form and followed a different way of abstraction. Hans Hofmann (1880-1966) started to paint his completely abstract works from the 1940s. His work shows a rigorous concern with pictorial structure and color relationships. Sonia Delaunay (1885-1979) worked as a multi-disciplinary artist and created beside textiles and theatre costumes, paintings composed of geometrical shapes in strong, vibrant colors.**

Artist	Label in Study	Painting	Date
**Willy Baumeister**	Ba. 1	Abstract composition	19? unknown
**Willy Baumeister**	Ba. 2	Monturi with red and blue	1953
**Willy Baumeister**	Ba. 3	Montaru 9	1953
**Willy Baumeister**	Ba. 4	Planar tension with red	1926
**Willy Baumeister**	Ba. 5	Happy day	1947
**Sonja Delaunay**	De. 1	Flamenco dancer	1916
**Hans Hoffmann**	Ho. 1	Golden spendor	1957
**Hans Hoffmann**	Ho. 2	Magnus opus	1962
**Hans Hoffmann**	Ho. 3	Veluti in speculum	1962
**Hans Hoffmann**	Ho. 4	Equipoise	1958
**Hans Hoffmann**	Ho. 5	Gloria mundi	1963
**Hans Hoffmann**	Ho. 6	The gate	1959-60
**Wassily Kandinsky**	Ka. 1	Composition 8	1923
**Wassily Kandinsky**	Ka. 2	Red oval	1920
**Wassily Kandinsky**	Ka. 3	Accent on rose	1926
**Wassily Kandinsky**	Ka. 4	Compensation rose	1933
**Wassily Kandinsky**	Ka. 5	Thirteen rectangles	1930
**Wassily Kandinsky**	Ka. 6	Upward	1929
**Paul Klee**	Kl. 1	Abstraction with reference to a flowering tree	1922
**Paul Klee**	Kl. 2	Before the blitz	1925
**Paul Klee**	Kl. 3	Cacodemonic	1923
**Paul Klee**	Kl. 4	Fire evening	1916
**Paul Klee**	Kl. 5	Fire, full moon	1929
**Paul Klee**	Kl. 6	The messenger of autumn	1933

Supplementary Table 2. **Artists, titles and dates of the 24 additional paintings used in the laterality preference experiment.**

Artist	Painting	Date
**Willy Baumeister**	Phantom and yellow table	1952
**Willy Baumeister**	Machine and red square	1926
**Willy Baumeister**	Phantom with red figure	1951
**Willy Baumeister**	Seaweed	1950
**Willy Baumeister**	The sculptor	1923
**Sonja Delaunay**	Rhythme colore	1952
**Hans Hoffmann**	Art like love is dedication	1965
**Hans Hoffmann**	Cathedral	1959
**Hans Hoffmann**	Pastorale	1958
**Hans Hoffmann**	Morning mist	1958
**Hans Hoffmann**	No 1	1953
**Hans Hoffmann**	Untitled	1949-50
**Wassily Kandinsky**	Reciprocal accords	1942
**Wassily Kandinsky**	Improvisation 4	1909
**Wassily Kandinsky**	Green composition	1923
**Wassily Kandinsky**	Composition 4	1911
**Paul Klee**	Small room in venice	1933
**Paul Klee**	City picture with red and green accents	1921
**Paul Klee**	Hamamet	1914
**Paul Klee**	Path and byways	1929
**Paul Klee**	Rising sun	1907
**Paul Klee**	Revolving house	1921
**Paul Klee**	Transparent	1921
**Paul Klee**	Color harmony	1924

Supplementary Table 3. **Data of the exiting survey. O: origin of participant E: Europe (Germany), C: China. Each column contains the data to one of the survey questions. See Supplementary Text1.**

O.	1. Interest?	2. Exhibitions?	3. Preferred style?	4. Like a. art?	5. Knew art?	6. Artists?	7. Fav. color?	8. Fav. task?
E	yes	1x	realism	it depends	0	0	no spec. color	1, 4
E	it depends	1x		yes	3	0	green	1
E	yes	1-2x	no	it depends	0	0	blue	2, 4
E	it depends	0	impressionism	so so	0	Klee	yellow	all
E	yes, very much	1x	abstract art	yes	2	0	green	4
E	yes, very much	6x	natural, realism, expressionism, surrealism	yes	5	Klee, Miro	rosy	1
E	yes	2-3x	impressionism	sometimes	0	Klee, Kandinsky	red	
E	yes	0	drawings, mandalas	not much	0	0	green	1, 4
E	yes	2x		sometimes	3	Kandinsky	dark red	1, 4
E	yes, very much	3x	surrealism, romantic	yes	2	0	yellow	4
E	yes	8x	expressionism, impressionism.	overall yes	0	0	violet	1
E	it depends	1x	I have to like it	it depends	2	1	all but pink	1
E	it depends	0	-	not much	0	0	0	1
E	it depends	1x	various	very much	0	0	yellow, red	1, 4
E	yes	1-2x	pop art, impressionism	it depends	4	Klee	blue	
E	yes	1x		yes	0	0	blue, red	1, 2
E	yes, very much	1x		no	5	Kandinsky	green	1
E	yes	2x		so so	0	0	green	1, 4
E	it depends					0	lavender, rosy	4
E	it depends	4x	surrealism	yes	0	0	no spec. color	1
C	no	no	no	no	0	0	dark yellow, red	4
C	it depends	1x	representational art	no	0	0	red	all
C	yes	1x	mangas		0	0	blue	
C					0	0	no	
C					0	0	pink, light yellow	
C		1x	impressionism	not really	0	0	blue	4
C		occasion.		no	0	0	no	
C	yes	4x 5x	Van Gogh	yes	0	0	orange	
C					0	0	blue	
C					0	0	rot	
C					0	0	light yellow	
C		1x		yes	0	0	lavender	
C		no	realistic		0	0	all colors	
C	yes	1x		yes	0	0	yellow & pink	
C	yes	1x			0	0	no spec. color	
C	yes	2x		yes	0	0	blue	
C	yes, very much	12x	realism	yes	0	0	orange	4
C	yes	24X	no specific	yes	0	0	red	4
C	yes, very much	1-2x	abstract	yes	0	0	red	
C	yes	2x	no specific	yes	0	0	pink	

Supplementary Figure 1. **Minimum distance analysis for each observer group. As in Figure 3A, bar length corresponds to the average minimum distance in CIE L*a* b* space between observer. The red line corresponds to the 1% cutoff, the pink line to the 5% cutoff. Minimum distances that are larger than this cutoff value can be said to be yielded by random color settings (light blue bars).**

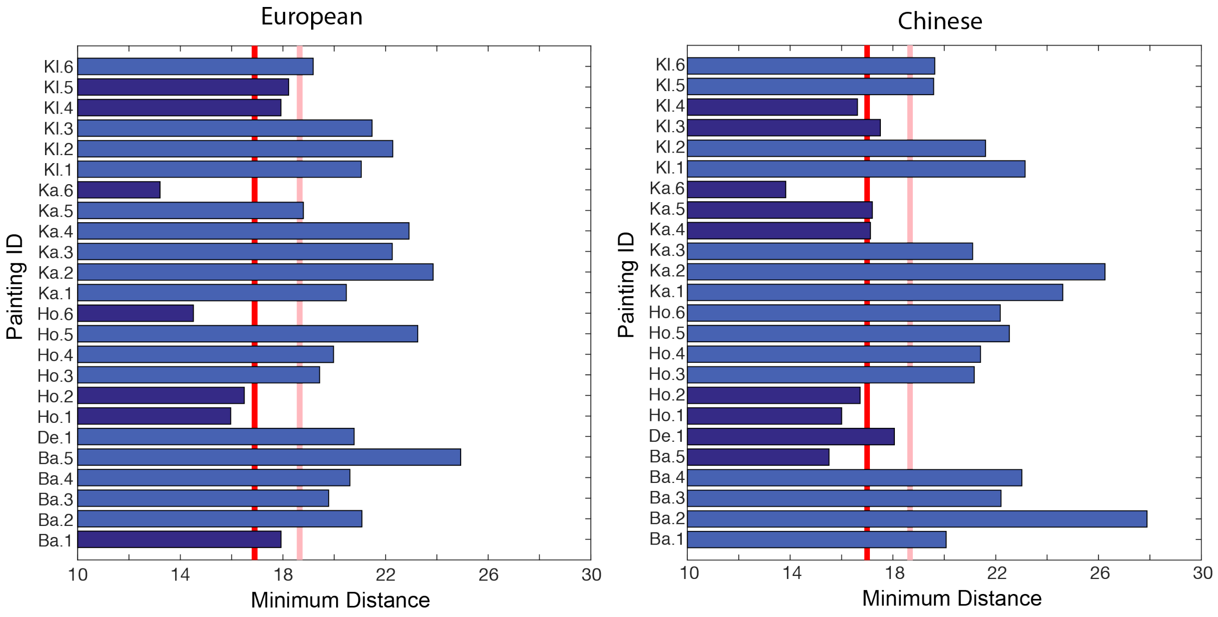

Supplementary Figure 2. **Pixel distributions of paint colors of the 24 JEPG images of the paintings and observers´ color settings in CIE L*a*b* space. The mean color of the target region is represented by a square. European participants are represented by circles, Chinese participants by diamonds. Individual paintings are referred to by the initials of the artists: Ba. for Baumeister, De. for Delaunay, Ho. for Hoffmann, Ka. for Kandinsky and Kl. for Klee and a painting number. Star symbols indicate the significance of the amount of clustering in the participants' setting distributions as assessed by minimum distance analysis at the 5% (*) and 1% (**) cutoff level respectively (see Figure 2). Corresponding circle and diamond symbols indicate the significance of the amount of clustering for European and Chinese Participants' respectively (Supplementary Figure 1). With the exception of Kl. 6, the pixel distributions do not show a general blue-yellow bias that is found for natural scenes and which are therefore commonly observed in paintings depicting nature by using a more blue-yellow palette (Tregilles & Webster, 2016). Overall, the CIE L*a*b* distribution of participants' target color settings quite often resembled the distribution of paint colors, potentially more than what was captured by the minimum distance analysis (Figure 2 & Supplementary Figure 1). Note, that these pixel distributions represent neglect important spatial aspects that are characteristic for each painting, such as the spatial distribution of paint colors, the specific setting of color contrasts at the borders of elements.**

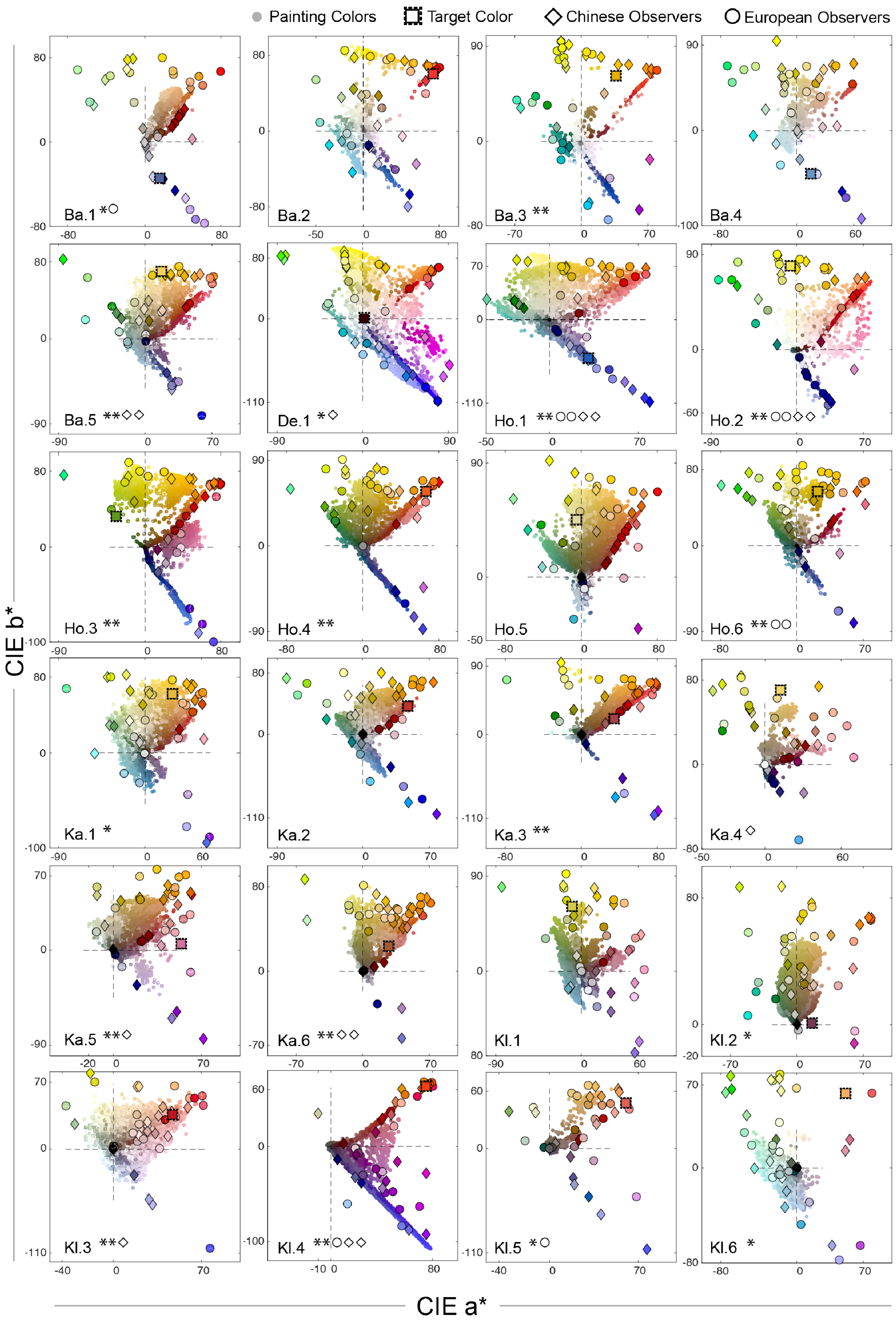

Painting Colors
Target Color
Chinese Observers
European Observers
CIE b*
CIE a*
Ba.1 *○
Ba.2
Ba.3 **
Ba.4
Ba.5 **◇◇
De.1 *◇
Ho.1 **○○◇◇
Ho.2 **○○◇◇
Ho.3 **
Ho.4 **
Ho.5
Ho.6 **○○
Ka.1 *
Ka.2
Ka.3 **
Ka.4 ◇
Ka.5 **◇
Ka.6 **◇◇
Kl.1
Kl.2 *
Kl.3 **◇
Kl.4 **○◇◇
Kl.5 *○
Kl.6 *

Supplementary Figure 3. **Target color settings and hue histograms. Participants' target settings (plotted as hue angle h°) as a function of painting ID. Target hue histograms on the right plot color target setting binned into eight hue angles, where each target color is weighted by its chroma: patches with low chroma will make the histogram 'grow' at a slower rate than those with high chroma values.**

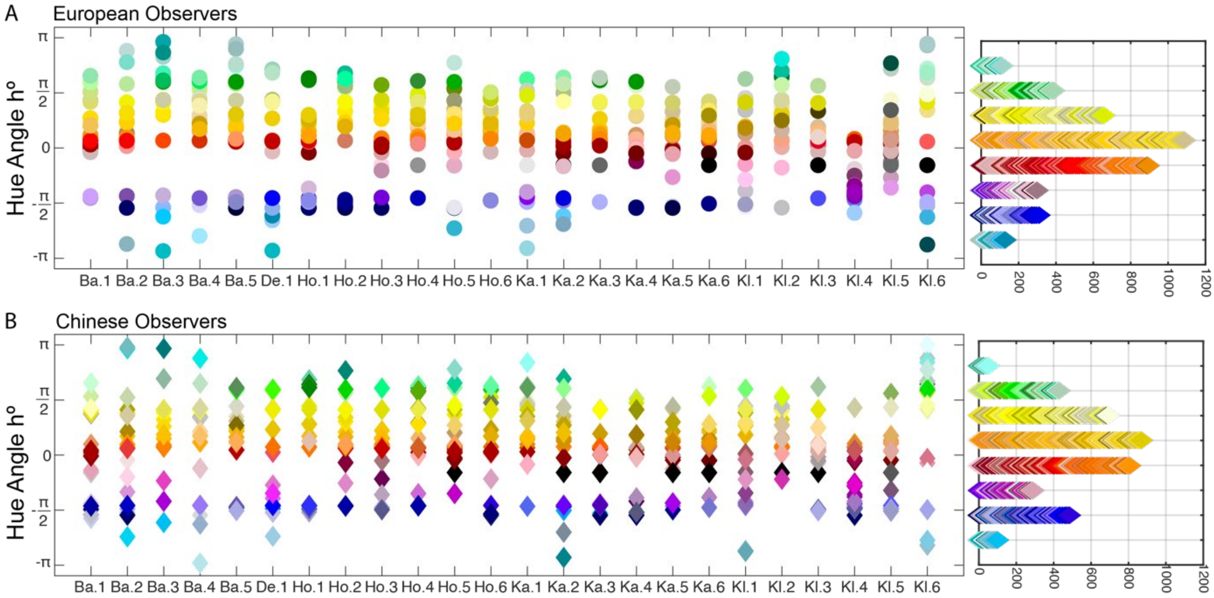

Supplementary Figure 4. **Target hue settings. Plotted are color target settings for the most frequently chosen hue category: for a given painting we plotted the target settings that belong to the 'winner' category along with the percentage of how many of the participants selected a hue from this category. Occasionally, the most frequent target hue setting fell into two or three categories. In those cases, the percentage applies to each of the categories, e.g. in panel B, 30% of participant settings fall into each of the three categories of Ka.1 (thus 90% of all participant settings fall into one of these three categories). Original target hues are plotted as framed symbols (roughly corresponding to their shape). Naturally, the frequency count is influenced by the location of the category boundaries. We set these roughly to correspond to shades of blue [$-2\pi/3$ $5\pi/6$] green [$3\pi/7$ $5\pi/6$] yellow-orange [$\pi/7$ $3\pi/7$] red [$-\pi/3$ $\pi/7$] and violet-purple [$-\pi/3$ $-2\pi/3$]. A. shows target hue settings for European Observers, B. for Chinese-, and C. for both observer groups combined. For most paintings one hue category dominated (except Ho. 1, Ho. 5 and Kl. 1 for European (Panel A), and Ho. 6 and Ka. 1 for Chinese participants (Panel B)). For some paintings, there was quite a high agreement among participants target hue settings, e.g. Kl. 3 (Panel A, 75% of observers chose a hue from the same category), whereas for others, target settings were more spread out, e.g. for Ba. 2 (Panel B, only 30 % of observers chose a hue from the same category). In general, the larger the color volume of a painting in CIE L*a*b* space the higher the amount of agreement for a given hue category among participants (R2=.26, p<0.013). Repeating this regression for each observer group we find that this relationship did not hold for European (R2=.12, p=0.09) but for Chinese observers (R2=0.22, p=0.019)). Overall, participants were quite successful at picking the 'right' target hue category for a painting.**

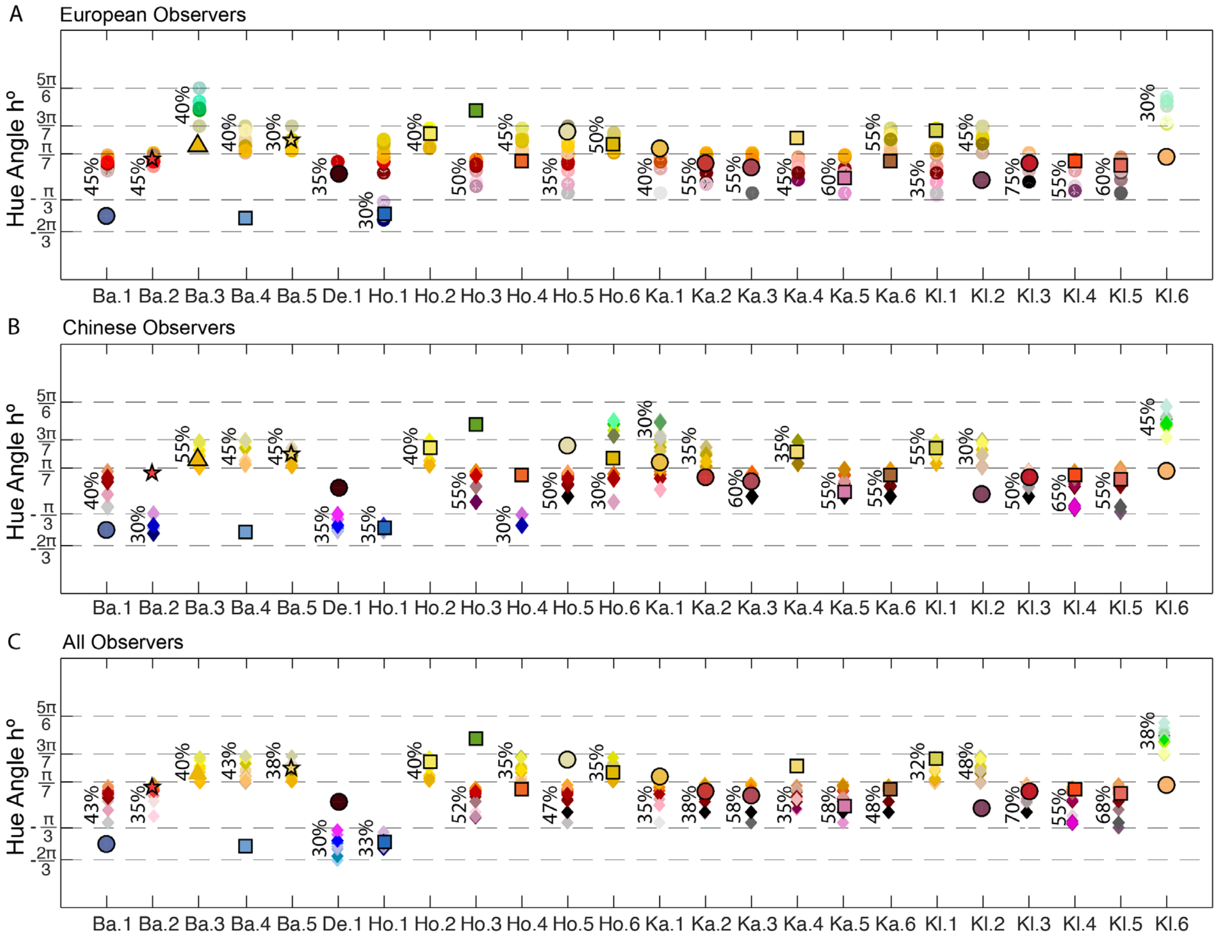

Supplementary Figure 5. Average luminance and chroma settings for the targets. Red diamonds show results for Chinese (PRC) observers, blue circles for Europeans (EU). The artist's target CIE L* luminance is plotted as open black squares in Panel A and the same symbols denote the artist's target _C*ab_ chroma values in Panel B. Overall, the standard error of the mean ranged between 3.63 (CIE L* for Ho. 6, European observers) and 8.34 (_C*ab_ for Kl. 3, European observers) and are omitted to avoid clutter. A 2 (groups) x 24 (paintings) Analysis of Variance revealed, that on average, Chinese observers tended to make their targets darker than European observers (CIE L*Ch= 46.75, CIE L*Eu= 51.66), yielding a main effect of observer group on luminance setting $F(1,912)=9.6$, $p<0.002$. There was no main effect of painting, and a significant interaction between the group and painting variable $F(23,912)=2.18$, $p<0.001$. Following up this interaction with a simple main effects analysis yielded no significant differences. There were also no effects of observer groups or painting ID on target chroma setting (Panel B). Panel C. shows the Weber Contrast of the target luminance setting with respect to the mean luminance of the painting. Overall, this contrast was significantly different from 0, $t(23)=4.947$, $p<0.0001$, suggesting that observers tended to choose target colors that exceeded the average luminance of the painting (mean contrast 0.388). These results are in line with findings by Schloss and Palmer (2011) who found that participants preferred color combinations that contrasted in lightness.

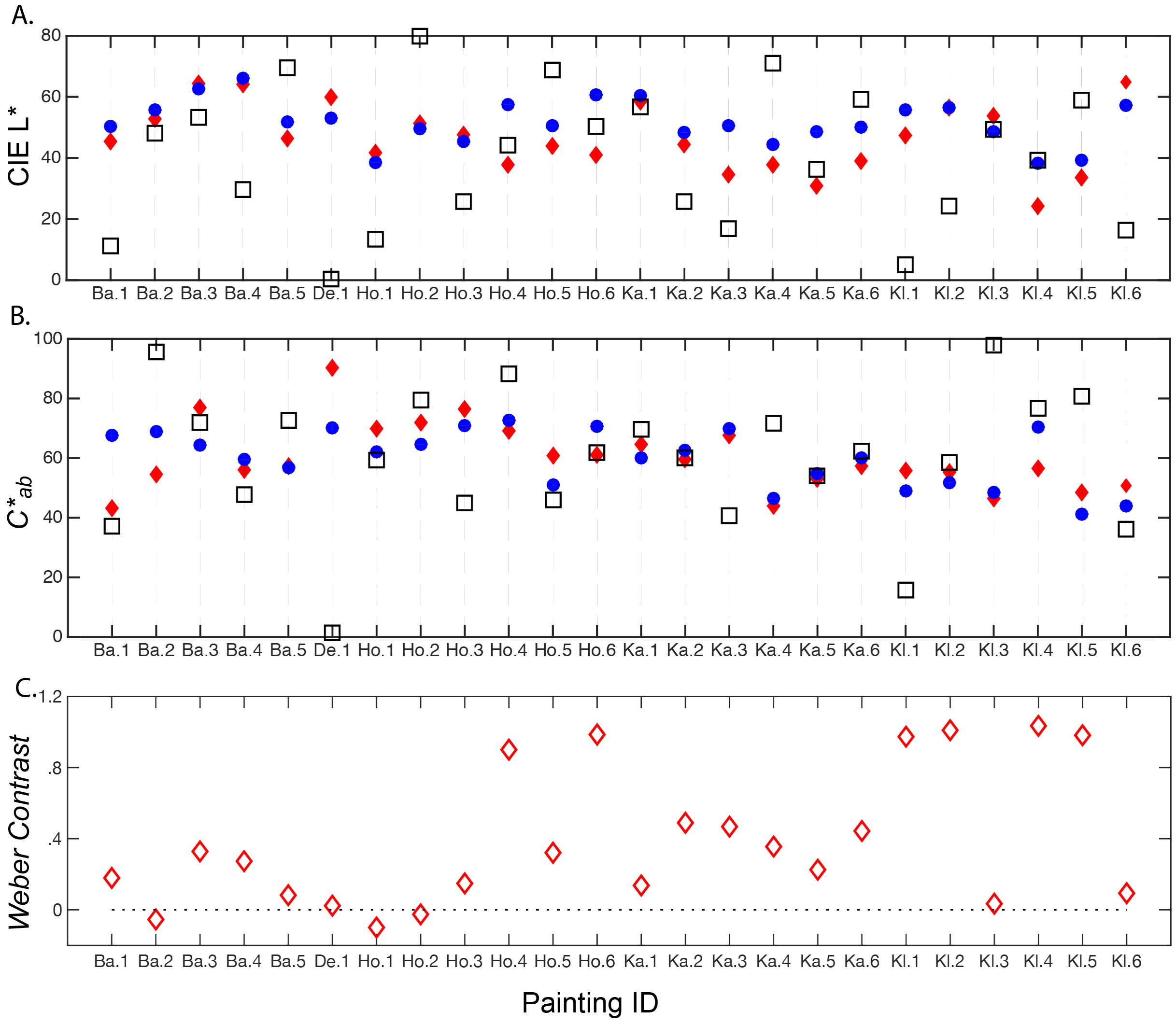

Supplementary Figure 6. **A. Proportion of target color settings inside CIE L*a*b* painting space. Bars show the proportion of target settings inside the volume consisting of the 20 CIE L*a*b* clusters for each painting. There was no difference between observer groups. Open squares are the number of settings that agreed with the target color of the artist. The latter did not occur very frequently. Red bars and symbols show results for Chinese observers, blue ones for Europeans. Bolded painting IDs mark paintings for which also the artist remained inside the paint palette with his target color. This was true for half (or 12) of the paintings. Note, that all of these counts are affected by the clustering method and/or the tolerance (see Analysis in main text). C. Shows that larger CIE L*a*b* painting volume (size) predicted the number of insiders for European observers, and D. illustrates that this was also true for Chinese observers.**

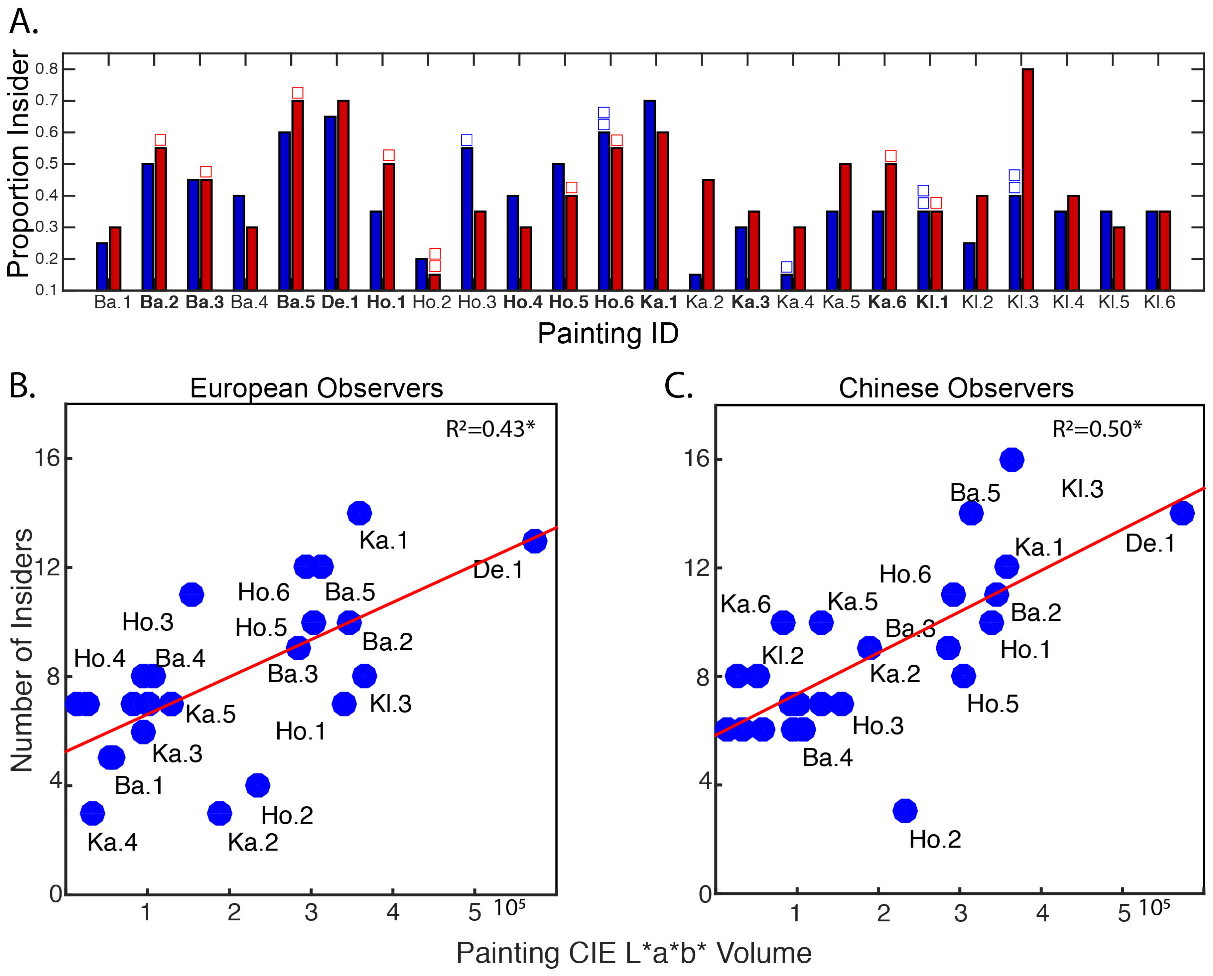

Supplementary Figure 7. **Proportion that the artist's target color was preferred. Red bars and symbols show results for Chinese observers, blue for Europeans. The red line denotes the 50% mark of equal preference for their own or the artist's color choice. The more bars extend above the red line, the stronger the preference for the artist's version, the more the bars extend below the red line, the stronger the preference for their own version. Overall, participants showed no clear preference for their own versus the artist's target color, indicating that they were quite content with their own color choices made for the target. Chinese observers tended to prefer the artist's color choice (yellow) for the big element in the painting "Happy day" of Baumeister (Ba. 5) and for the small circle in the painting "Cacodemonic" (Kl. 3) Klee's choice of red. European participants tended to agree that the artist's yellow was a better for the target in Baumeister's Montaru (Ba. 3) and the color violet for the painting "The blitz" of Klee (Kl. 2). Chinese participants tended not to "like" blue for the target in Baumeister's "Planar tension with red" (Ba. 4) or orange for the target in Klee's "The messenger of autumn" (Kl. 6). For Kl. 2, observer groups tended to differ most in their preferences (zDiff=2.51). Largest preference differences occurred for Ba. 3: zEu=2.12; Ba. 4: zCh=-2.12; Kl. 2: zEu=2.5; Kl. 3: zCh=2.12; Kl. 6: zCh=-2.12, however with a cutoff value of z>3 (to correct for Type I error inflation) these differences did not reach statistical significance.**

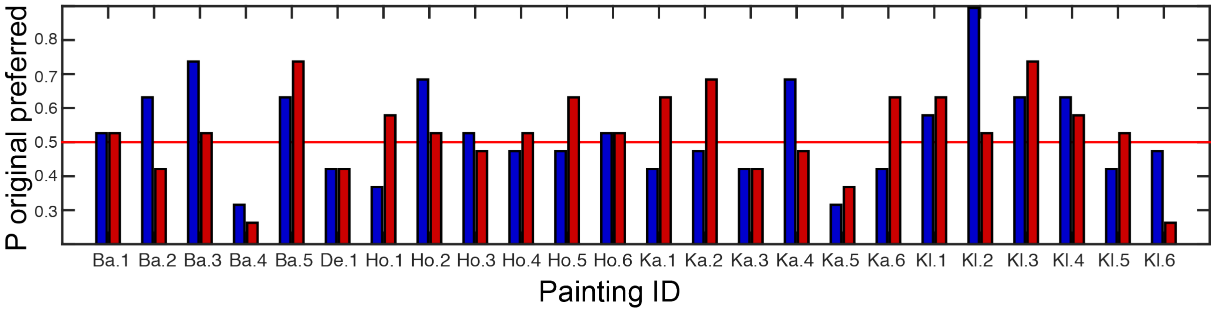

Supplementary Figure 8**. Shown are the average relative sizes of balance points (diameter of the balance point divided by the smaller dimension of the painting) for each painting and both observer groups (Chinese observers: red diamonds, European observers: blue circles). Error bars are 1 standard error of the mean. Overall observers were able to do this task, choosing the 'no balance point' option only on 17.4% of the trials (red and blue bars). Chinese observers used this option in 17%, and European in 18 % of the trials more or less evenly for all 24 paintings. The settings for circle size, which indicated the strength of the balance point, varied significantly across paintings and observer groups (dot and diamond symbols). A 2 (observer group) x 24 (painting ID) ANOVA on relative probe size (dot probe diameter/divided by the smaller dimension of the painting, both measured in degrees visual angle) yielded a significant main effect of painting ID F(24,1)=5.36, p<0.0001 and of observer group F(2,1)=17.44, p<0.0001. There was no significant interaction. Overall, Chinese observers tended to judge their chosen balance point as more important - making the dot probe slightly larger than European observers (mean_relative_sizeChinese=21.53, mean_relative sizeEuropean=16.66%). Posthoc analysis of the main effect for painting ID using Bonferroni correction (at alpha set to 0.05) showed that the size of the dot probe for Ba.3 was significantly larger than in all other paintings. The paintings Kl. 2 and Kl. 3 yielded the smallest dot probe sizes: Kl. 2<Ba. 2 and Ka. 1, and Kl. 3<Ba. 2, Ho. 1, Ho. 6 and Ka. 1.**

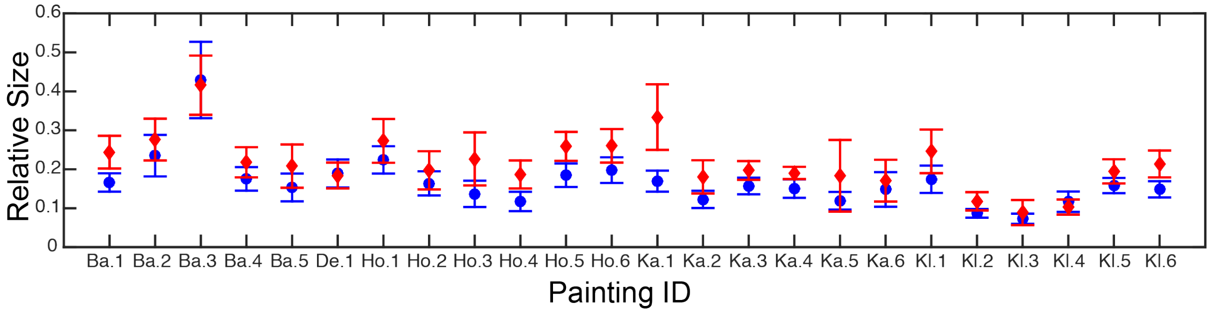

Supplementary Figure 9. **Balance point settings of all 40 observers. Shown are x and y coordinates of the circle probe setting for each observer (Chinese: red diamonds, European: blue circles) and painting. Black dashed lines bisect each painting horizontally and vertically. All images and data were scaled to fit the panel dimension, original aspect ratios (height/width) are given as insets. The central gray circle (also scaled along with the painting) has a radius of 2 degrees visual angle and approximately corresponds to the central region of each painting. In this representation, a painting that is larger in height than in width will look compressed along the vertical dimension (e.g. Ba. 1) and the central gray area is shaped like a horizontal ellipsoid. Also remember, that in the experiments all paintings were scaled along their largest extend (width or height) to approximately 20 deg. visual angle. To help reading this plot we insert aspect ratios (width/height) of the paintings as inset to each panel. We plot this to help the reader to appreciate that the balance point location tended to be outside the geometric center of the painting. This figure illustrates that in general, observers use the painting structure to estimate the balance point. The three images Ka. 2, Ka. 5 and Kl. 4 were perceived as most centrally "balanced" and most observers placed balance points into the corresponding center of the rectangular element. For the paintings Ho. 5, Ho. 6 and Kl. 2, which are more symmetrical along the vertical axis, also observers' balance points were distributed along the vertical.**

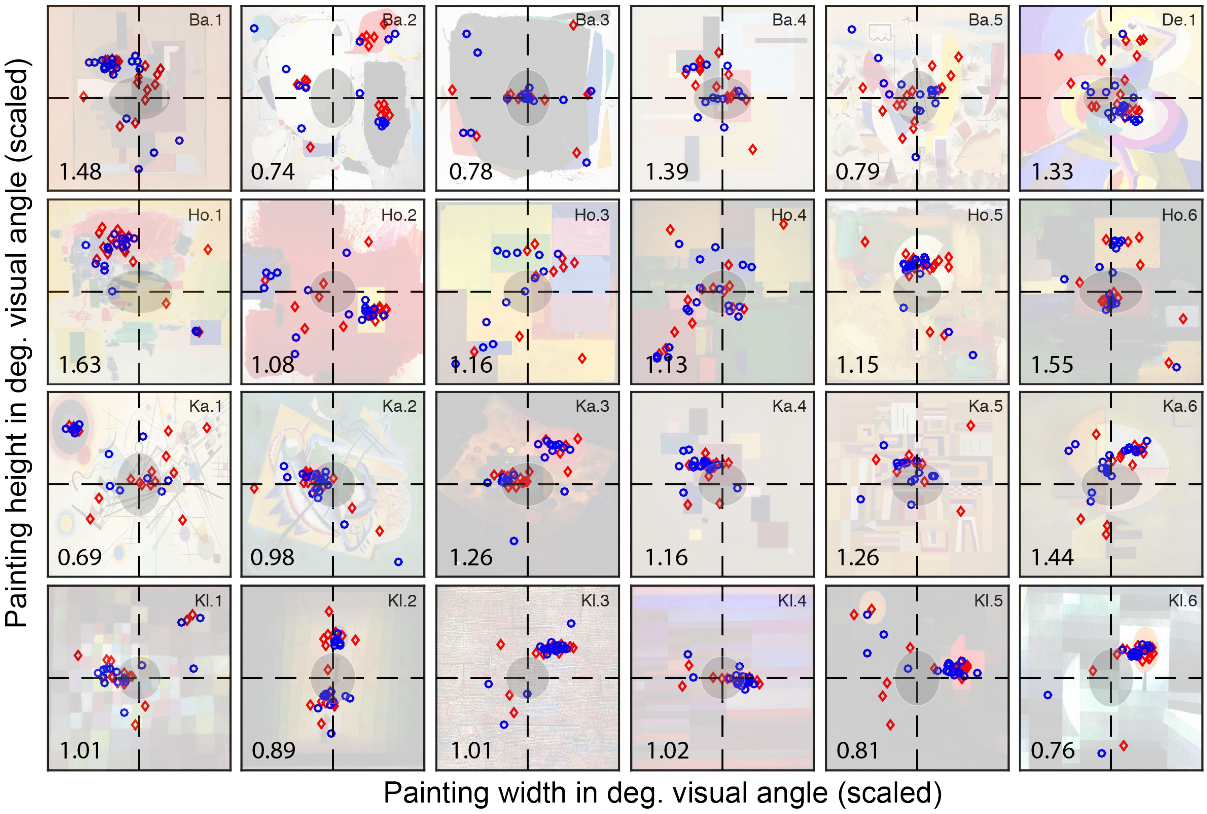

Supplementary Figure 10. **Proportion that the flipped, i.e. left-right mirror reversed version of the painting was preferred. Gray bars in the top row show preferences across both observer groups for the 24 familiar paintings used in experiments 1-3, blue bars denote orientation preferences for the additional 24 unfamiliar paintings. The red line denotes the 50% mark of equal preference for the flipped as the original orientation. Bars below this line indicate a preference for the artist intended orientation of the painting. Significant differences from the 50% mark are marked with an asterisk (p <0.0021 (corresponds to a z cutoff of z>3), alpha level .05, two-tailed. corrected for multiple (24) comparisons).**

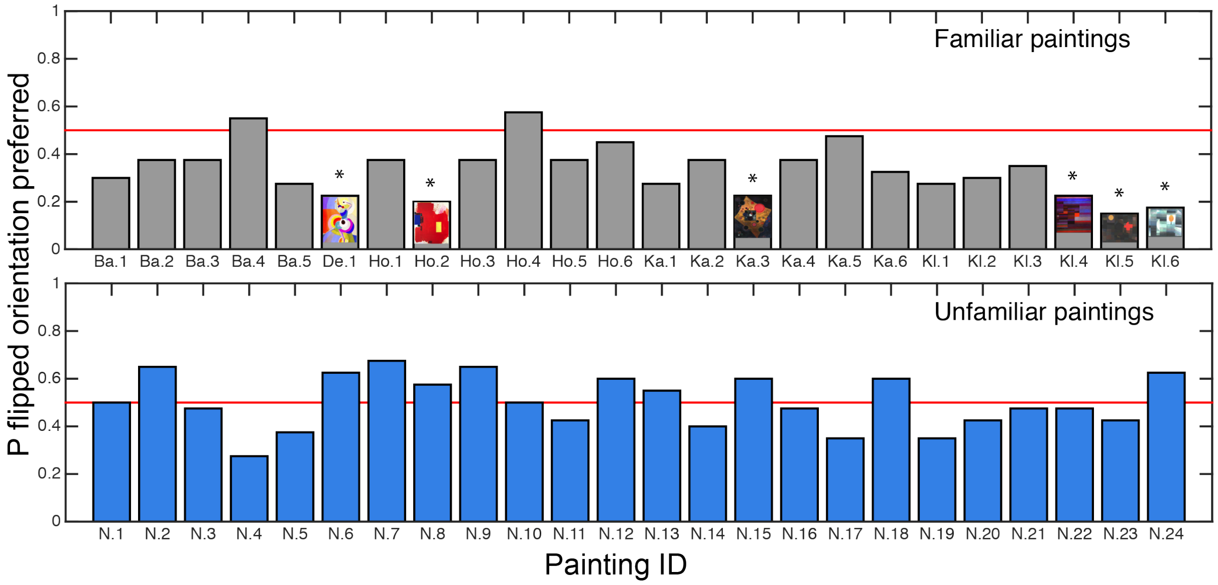

Supplementary Figure 11. **Relationship between perceived center of gravity and orientation preference. Along the x-axis we plot the relative x displacements (dividing the distance in x from the painting center by the size of x dimension of the painting, in degrees of visual angle. Larger negative values mean a larger leftward distance of the circle probe to the center, larger positive values mean a larger rightward distance of the dot probe to the center. On the y-axis we plot the proportion of the average side preference indicated by the observers. Larger positive values mean that the original was preferred, larger negative values mean that the flipped version was preferred. Values close to 0 indicate no preference. The relationship between side preference and dot distance was significant. The shift of the data towards 'prefer original orientation' suggests a memory effect. The more a balance point was perceived to be on the right, the more the original orientation of the painting was preferred. The more leftward the balance point was perceived to be in the original painting, the more observers preferred the flipped version. This result was quite surprising. (R^2=.29, p=0.02).**

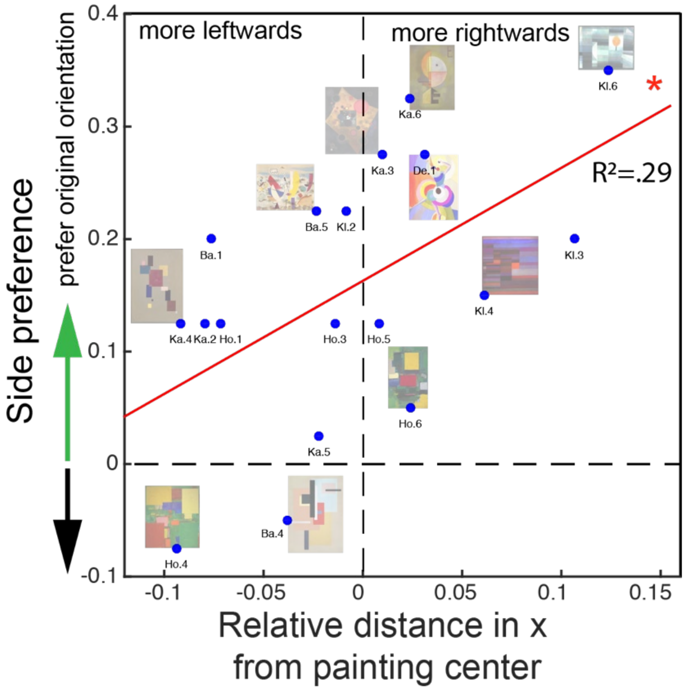

Supplement: Supplemental material for Kandinsky or Me? How Free Is the Eye of the Beholder in Abstract Art? [file Supplemental_Material.pdf]
